# Baseline Expression of Immune Gene Modules in Blood is Associated With Primary Response to Anti-TNF Therapy in Crohn’s Disease Patients

**DOI:** 10.1093/ecco-jcc/jjad166

**Published:** 2023-09-30

**Authors:** Benjamin Y H Bai, Mark Reppell, Nizar Smaoui, Jeffrey F Waring, Valerie Pivorunas, Heath Guay, Simeng Lin, Neil Chanchlani, Claire Bewshea, James R Goodhand, Nicholas A Kennedy, Tariq Ahmad, Carl A Anderson, UK Inflammatory Bowel Disease Pharmacogenetics Study Group, Vinod Patel, Zia Mazhar, Rebecca Saich, Ben Colleypriest, Tony C Tham, Tariq H Iqbal, Vishal Kaushik, Senthil Murugesan, Salil Singhi, Sean Weaver, Cathryn Preston, Assad Butt, Melissa Smith, Dharamveer Basude, Amanda Beale, Sarah Langlands, Natalie Direkze, Miles Parkes, Franco Torrente, Juan De La Revella Negro, Chris Ewen MacDonald, Stephen M Evans, Anton V J Gunasekera, Alka Thakur, David Elphick, Achuth Shenoy, Chuka U Nwokolo, Anjan Dhar, Andrew T Cole, Anurag Agrawal, Stephen Bridger, Julie Doherty, Sheldon C Cooper, Shanika de Silva, Craig Mowat, Phillip Mayhead, Charlie Lees, Gareth Jones, Tariq Ahmad, James W Hart, Daniel R Gaya, Richard K Russell, Lisa Gervais, Paul Dunckley, Tariq Mahmood, Paul J R Banim, Sunil Sonwalkar, Deb Ghosh, Rosemary H Phillips, Amer Azaz, Shaji Sebastian, Richard Shenderey, Lawrence Armstrong, Claire Bell, Radhakrishnan Hariraj, Helen Matthews, Hasnain Jafferbhoy, Christian P Selinger, Veena Zamvar, John S De Caestecker, Anne Willmott, Richard Miller, Palani Sathish Babu, Christos Tzivinikos, Stuart L Bloom, Guy Chung-Faye, Nicholas M Croft, John M E Fell, Marcus Harbord, Ailsa Hart, Ben Hope, Peter M Irving, James O Lindsay, Joel E Mawdsley, Alistair McNair, Kevin J Monahan, Charles D Murray, Timothy Orchard, Thankam Paul, Richard Pollok, Neil Shah, Sonia Bouri, Matt W Johnson, Anita Modi, Kasamu Dawa Kabiru, B K Baburajan, Bim Bhaduri, Andrew Adebayo Fagbemi, Scott Levison, Jimmy K Limdi, Gill Watts, Stephen Foley, Arvind Ramadas, George MacFaul, John Mansfield, Leonie Grellier, Mary-Anne Morris, Mark Tremelling, Chris Hawkey, Sian Kirkham, Charles P J Charlton, Astor Rodrigues, Alison Simmons, Stephen J Lewis, Jonathon Snook, Mark Tighe, Patrick M Goggin, Aminda N De Silva, Simon Lal, Mark S Smith, Simon Panter, Fraser Cummings, Suranga Dharmisari, Martyn Carter, David Watts, Zahid Mahmood, Bruce McLain, Sandip Sen, Anna J Pigott, David Hobday, Emma Wesley, Richard Johnston, Cathryn Edwards, John Beckly, Deven Vani, Subramaniam Ramakrishnan, Rakesh Chaudhary, Nigel J Trudgill, Rachel Cooney, Andy Bell, Neeraj Prasad, John N Gordon, Matthew J Brookes, Andy Li, Stephen Gore

**Affiliations:** Genomics of Inflammation and Immunity Group, Wellcome Sanger Institute, Hinxton, UK; Postgraduate School of Life Sciences, University of Cambridge, Cambridge, UK; AbbVie Inc., Chicago, IL, USA; AbbVie Inc., Chicago, IL, USA; AbbVie Inc., Chicago, IL, USA; AbbVie Inc., Chicago, IL, USA; AbbVie Inc., Chicago, IL, USA; Department of Gastroenterology, Royal Devon University Healthcare NHS Foundation Trust, Exeter, UK; Exeter Inflammatory Bowel Disease and Pharmacogenetics Research Group, University of Exeter, Exeter, UK; Department of Gastroenterology, Royal Devon University Healthcare NHS Foundation Trust, Exeter, UK; Exeter Inflammatory Bowel Disease and Pharmacogenetics Research Group, University of Exeter, Exeter, UK; Exeter Inflammatory Bowel Disease and Pharmacogenetics Research Group, University of Exeter, Exeter, UK; Department of Gastroenterology, Royal Devon University Healthcare NHS Foundation Trust, Exeter, UK; Exeter Inflammatory Bowel Disease and Pharmacogenetics Research Group, University of Exeter, Exeter, UK; Department of Gastroenterology, Royal Devon University Healthcare NHS Foundation Trust, Exeter, UK; Exeter Inflammatory Bowel Disease and Pharmacogenetics Research Group, University of Exeter, Exeter, UK; Department of Gastroenterology, Royal Devon University Healthcare NHS Foundation Trust, Exeter, UK; Exeter Inflammatory Bowel Disease and Pharmacogenetics Research Group, University of Exeter, Exeter, UK; Genomics of Inflammation and Immunity Group, Wellcome Sanger Institute, Hinxton, UK; Tameside Hospital NHS Foundation Trust, Ashton U Lyne; Basildon and Thurrock University Hospitals NHS Foundation Trust, Basildon; Hampshire Hospitals NHS Foundation Trust, Basingstoke; Royal United Hospital, Bath; Ulster Hospital, Belfast; University Hospital's Birmingham NHS Foundation Trust, Birmingham; East Lancashire NHS Teaching Trust, Blackburn; Blackpool Teaching Hospitals NHS Foundation Trust, Blackpool; Royal Bournemouth Hospital, Bournemouth; Royal Bournemouth Hospital, Bournemouth; Bradford Teaching Hospitals Foundation Trust - (St Lukes Hospital &Bradford Royal Infirmary), Bradford; Brighton and Sussex University Hospitals NHS Trust, Brighton; Brighton and Sussex University Hospitals NHS Trust, Brighton; University Hospitals Bristol NHS Foundation Trust, Bristol; University Hospitals Bristol NHS Foundation Trust, Bristol; Frimley Park Hospital NHS Foundation Trust, Camberley; Frimley Park Hospital NHS Foundation Trust, Camberley; Cambridge University Hospitals NHS Foundation Trust, Cambridge; Cambridge University Hospitals NHS Foundation Trust, Cambridge; Cambridge University Hospitals NHS Foundation Trust, Cambridge; North Cumbria University Hospitals NHS Trust, Carlisle; Ashford & St Peter's Hospitals NHS Foundation Trust, Chertsey; St Peter's Hospital, Chertsey; Ashford & St Peter's Hospitals NHS Foundation Trust, Chertsey; Chesterfield Royal NHS Foundation Trust, Chesterfield; Colchester Hospital University NHS Foundation Trust, Colchester; University Hospitals Coventry and Warwickshire NHS Trust, Coventry; County Durham and Darlington NHS Foundation Trust, Darlington; Derby Hospital NHS Foundation NHS Trust, Derby; Doncaster and Bassetlaw Hospitals NHS Foundation Trust, Doncaster; Dorset County Hospital NHS Foundation Trust, Dorchester; Dorset County Hospitals Foundation Trust, Dorchester; Dudley Group NHS Foundation Trust, Dudley; Dudley Group NHS Foundation Trust, Dudley; Ninewells Hospital & Medical School, Dundee; East Sussex Healthcare Trust, Eastborne; NHS Lothian, Edinburgh; NHS Lothian, Edinburgh; Royal Devon and Exeter NHS Foundation Trust, Exeter; Royal Devon and Exeter NHS Foundation Trust, Exeter; Glasgow Royal Infirmary, Glasgow; Royal Hospital for Children, Glasgow; Royal Hospital for Children, Glasgow; Gloucestershire Hospitals NHS Trust, Gloucester; United Lincolnshire Hospitals NHS Trust, Grantham; James Paget University Hospitals NHS Foundation Trust, Great Yarmouth; Calderdale and Huddersfield NHS Trust, Halifax; Princess Alexandra Hospital NHS Trust, Harlow; Princess Alexandra Hospital NHS Trust, Harlow; Hull and East Yorkshire NHS Trust, Hull; Hull and East Yorkshire NHS Trust, Hull; Airedale NHS Foundation Trust, Keighley; Crosshouse Hospital, Kilmarnock; Crosshouse Hospital, Kilmarnock; The Queen Elizabeth Hospital NHS Foundation Trust, Kings Lynn; Kingston Hospital NHS Trust, Kingston upon Thames; NHS Fife, Kirkcaldy; Leeds Teaching Hospitals NHS Trust, Leeds; Leeds Teaching Hospitals NHS Trust, Leeds; University Hospitals of Leicester NHS Trust, Leicester; University Hospitals of Leicester NHS Trust, Leicester; Mid Cheshire Hospitals NHS Foundation Trust, Leighton; United Lincolnshire Hospitals NHS Trust, Lincoln; Alder Hey Childrens Hospital, Liverpool; University College London Hospitals NHS Foundation Trust, London; Kings College Hospital NHS Foundation Trust, London; Royal London Childrens Hospital, Barts Health NHS Trust, London; Chelsea & Westminster Hospital, London; Chelsea and Westminster Hospital NHS Foundation, London; North West London Hospitals NHS Trust, London; Kings College Hospital NHS Foundation Trust, London; Guys & St Thomas' NHS Foundation Trust, London; Barts and The London NHS Trust, London; Guy's and St Thomas' NHS trust, London; Lewisham and Greenwich Healthcare NHS Trust, London; Chelsea and Westminster Hospital NHS Foundation, London; Royal Free London NHS Foundation Trust, London; Imperial College Healthcare NHS Trust, London; St George's Healthcare NHS Trust, London; St George's Healthcare NHS Trust, London; Great Ormond Street Hospital for Children NHS Foundation Trust, London; North West London Hospitals NHS Trust, London; The Luton & Dunstable University Hospital, Luton; Luton and Dunstable Hospital Foundation Trust, Luton; The Luton & Dunstable University Hospital, Luton; Maidstone and Tunbridge Wells NHS Trust, Maidstone; Maidstone and Tunbridge Wells NHS Trust, Maidstone; Manchester University Hospitals NHS Foundation Trust, Manchester; Central Manchester University Hospitals NHS Foundation Trust, Manchester; The Pennine Acute Hospitals NHS Trust, Manchester; Manchester University NHS Foundation Trust, Wythenshawe Hospital, Manchester; Sherwood Forest Hospitals NHS Foundation Trust, Mansfield; South Tees Hospital NHS Foundation Trust, Middlesbrough; Milton Keynes Hospital NHS Foundation Trust, Milton Keynes; Newcastle Upon Tyne Hospital Trust, Newcastle; Isle of Wight NHS Foundation Trust, Newport; Norfolk & Norwich University Hospital NHS Foundation Trust, Norwich; Norfolk & Norwich University Hospital NHS Foundation Trust, Norwich; Nottingham University Hospitals NHS Trust, Nottingham; Nottingham University Hospitals NHS Trust, Nottingham; Nottingham University Hospitals NHS Trust, Nottingham; Oxford University Hospitals NHS Foundation Trust, Oxford; Oxford University Hospitals NHS Trust, Oxford; Plymouth Hospitals NHS Trust, Plymouth; Poole Hospital NHS Foundation Trust, Poole; Poole Hospital NHS Foundation Trust, Poole; Portsmouth Hospitals NHS Trust, Portsmouth; Royal Berkshire NHS Foundation Trust, Reading; Salford Royal NHS Foundation Trust, Salford; Shrewsbury and Telford Hospital NHS Trust, Shrewsbury; South Tyneside NHS Foundation Trust, South Shields; Southampton University Hospitals NHS Trust, Southampton; Southampton University Hospitals NHS Trust, Southampton; East and North Herts NHS Trust, Stevenage; NHS Forth Valley, Stirling; Stockport NHS foundation Trust, Stockport; North Tees and Hartlepool NHS Foundation Trust, Stockton; University Hospitals of North Staffordshire, Stoke-on Trent; University Hospitals of North Midlands NHS Trust, Stoke-on Trent; City Hospitals Sunderland NHS Foundation Trust, Sunderland; Taunton and Somerset NHS Foundation Trust, Taunton; South Devon Healthcare NHS Foundation Trust, Torquay; South Devon Healthcare NHS Foundation Trust, Torquay; Royal Cornwall Hospitals NHS Trust, Truro; Mid Yorkshire Hospitals NHS Trust, Wakefield; Warrington& Halton NHS Foundation, Warrington; West Hertfordshire Hospitals NHS Trust, Watford; Sandwell and West Birmingham Hospitals NHS Trust, West Bromwich; Sandwell and West Birmingham Hospitals NHS Trust, West Bromwich; Weston Area Health NHS Trust, Weston-Super-Mare; Royal Albert Edward Infirmary, Wrightington, Wigan & Leigh NHS Foundation Trust, Wigan; Hampshire Hospitals NHS Foundation Trust, Winchester; Royal Wolverhampton Hospitals NHS Trust, Wolverhampton; Western Sussex Hospitals NHS Trust, Worthing; Yeovil District Hospital NHS Foundation Trust, Yeovil

**Keywords:** Anti-TNF, Crohn’s disease, transcriptomic biomarkers

## Abstract

**Background and Aims:**

Anti-tumour necrosis factor [anti-TNF] therapy is widely used for the treatment of inflammatory bowel disease, yet many patients are primary non-responders, failing to respond to induction therapy. We aimed to identify blood gene expression differences between primary responders and primary non-responders to anti-TNF monoclonal antibodies [infliximab and adalimumab], and to predict response status from blood gene expression and clinical data.

**Methods:**

The Personalised Anti-TNF Therapy in Crohn’s Disease [PANTS] study is a UK-wide prospective observational cohort study of anti-TNF therapy outcome in anti-TNF-naive Crohn’s disease patients [ClinicalTrials.gov identifier: NCT03088449]. Blood gene expression in 324 unique patients was measured by RNA-sequencing at baseline [week 0], and at weeks 14, 30, and 54 after treatment initiation [total sample size = 814].

**Results:**

After adjusting for clinical covariates and estimated blood cell composition, baseline expression of major histocompatibility complex, antigen presentation, myeloid cell enriched receptor, and other innate immune gene modules was significantly higher in anti-TNF responders vs non-responders. Expression changes from baseline to week 14 were generally of consistent direction but greater magnitude [i.e. amplified] in responders, but interferon-related genes were upregulated uniquely in non-responders. Expression differences between responders and non-responders observed at week 14 were maintained at weeks 30 and 54. Prediction of response status from baseline clinical data, cell composition, and module expression was poor.

**Conclusions:**

Baseline gene module expression was associated with primary response to anti-TNF therapy in PANTS patients. However, these baseline expression differences did not predict response with sufficient sensitivity for clinical use.

## 1. Introduction

Crohn’s disease [CD] is a chronic immune-mediated inflammatory disease [IMID] of the gastrointestinal tract. Along with ulcerative colitis [UC], it is one of the two main forms of inflammatory bowel disease [IBD]. The development of anti-tumour necrosis factor [TNF] biological therapies has revolutionized patient care for CD and a number of other IMIDs over the last two decades. Two major anti-TNF drugs, infliximab and adalimumab, are IgG1 monoclonal antibodies that bind both soluble and transmembrane TNF, inhibiting their interactions with TNF receptors.^1,2^ Two main mechanisms of action have been proposed: induction of CD4^+^ T cell apoptosis in the gut mucosa by inhibiting the TNF–TNFR2 interaction; and binding of the antibody tail [Fc region] of the drug to Fc receptors on monocytes, inducing their differentiation into wound-healing M2 macrophages.^3,4^

Unfortunately, anti-TNF therapy is not always effective at treating IBD. Various types of treatment failure can occur: primary non-response [PNR] within the induction period [the first 12–14 weeks for infliximab and adalimumab], secondary loss of response [LOR] during maintenance therapy after an initial response, failure to achieve remission after the treatment course, or adverse events that lead to treatment discontinuation.^5^ For IBD patients, the incidence of PNR is 10–40%, and the incidence of secondary LOR among initial responders is 24–46% in the first year of treatment.^6–8^ The ability to predict PNR and LOR could help guide changes in treatment regimens, such as dose intensification or switching to a drug class with a different mechanism of action.^2,6^ Reliable baseline prediction would be especially valuable, allowing stratification of patients to effective therapies from treatment initiation, minimizing healthcare costs and patient burden.

Clinical variables reported to be associated with anti-TNF response include age, disease duration, body mass index [BMI], smoking, C-reactive protein [CRP] levels, faecal calprotectin levels, serum drug concentrations, and anti-drug antibody concentrations.^7,9–13^ In the Personalised Anti-TNF Therapy in Crohn’s Disease [PANTS] study, the largest study of infliximab and adalimumab response in CD patients to date [enrolment *n* = 1610], baseline obesity, smoking, and greater disease activity were associated with low serum drug concentration after induction. A low drug concentration was in turn associated with PNR and non-remission, suggesting immunogenicity may be mediating treatment failure by increasing the drug clearance rate.^8^

Multiple studies have also reported transcriptomic predictors for anti-TNF response.^12–20^ One such example is *TREM1* expression, identified as a marker of anti-TNF response in different studies with inconsistent directions of effect. Gaujoux et al.^16^ found *TREM1* expression was lower in gut biopsies from infliximab responders than in non-responders [total cohort size *n* = 72], but higher in responders in a separate cohort measuring baseline whole blood expression [*n* = 22]. By contrast, Verstockt et al.^18^ reported lower *TREM1* expression in responders to infliximab and adalimumab in both baseline gut biopsies [*n* = 44] and baseline whole blood [*n* = 54]. Proposed reasons for the discrepancy include false positives due to small sample sizes, differences in patient ethnicity, and differing definitions of response.^12,21^ In general, small sample sizes, and variation among studies in analysis methods, anti-TNF drug, response definition, tissues sampled, and disease subtype make a consensus hard to establish. Few markers for anti-TNF response of any type, clinical or transcriptomic, have been validated in independent studies, and none has yet been translated to routine clinical practice.^13^

To identify novel transcriptomic associations with primary response to anti-TNF therapy, we generated longitudinal RNA-sequencing [RNA-seq] data from peripheral blood samples taken from a subset of the PANTS cohort (182 primary response [PR], 142 PNR) during the first year of follow-up. Differential gene expression [DGE] between primary responders and non-responders was performed at baseline [week 0], post-induction [week 14], and during maintenance [weeks 30 and 54]. We detected differences in gene module expression that may reflect differences in disease characteristics or severity that influence risk of primary non-response. As this is one of the largest datasets currently available for assessing transcriptomic associations with anti-TNF response in IBD, we also examined the significance of previously reported transcriptomic markers from the literature. Finally, we evaluated the utility of measuring module expression for prediction of primary response status.

## 2. Materials and Methods

### 2.1. Study design

PANTS is a UK-wide, multicentre, prospective observational cohort study reporting the treatment failure rates of the anti-TNF drugs infliximab (originator, Remicade [Merck Sharp & Dohme] and biosimilar, CT-P13 [Celltrion]), and adalimumab (Humira [AbbVie]) in 1610 anti-TNF naive CD patients. The study design has been described in detail previously.^8,22^ In brief, patients were recruited at the time of first anti-TNF exposure between February 2013 and June 2016, and evaluated for 12 months or until drug withdrawal. Eligible patients were aged ≥6 years with evidence of active luminal CD involving the colon and/or small intestine. Four major study visits were scheduled at week 0 [baseline], week 14 [post-induction], week 30, and week 54. Additional visits were scheduled at treatment failure or study exit. At baseline, clinical and demographic data were recorded, including sex, ethnicity, BMI, smoking status, age at diagnosis, disease duration, Montreal classification, prior medical and drug history, and previous CD-related surgeries. At every visit, disease activity score, weight, current therapy, and adverse events were recorded.^8^

### 2.2. RNA-seq sample selection

A subset of PANTS patients was selected for RNA-seq, with the inclusion criteria: age ≥ 16 years; and baseline CRP ≥ 4 mg/L and/or baseline calprotectin > 100 µg/g. The target sample size was 200 patients on infliximab and 200 patients on adalimumab, with an even split between PR and PNR within each drug group. PR and PNR were defined based on patient outcome criteria from Kennedy et al.:^8^

- *Primary non-response [assessed at week 14]*: exit before week 14 because of treatment failure [including resectional IBD surgery] or corticosteroid use at week 14 [new prescriptions or if previous dose had not been stopped]. Patients whose CRP did not decrease to ≤3 mg/L or by ≥50% from baseline [week 0], and whose Harvey–Bradshaw index [HBI] score did not decrease to ≤4 points or by ≥3 points from baseline were also classified as having a primary non-response.
- *Primary response [assessed at week 14]:* decrease in CRP to ≤3 mg/L or by ≥50% from baseline [week 0], and a decrease in HBI to ≤4 points or by ≥3 points from baseline.
- *Remission [assessed at weeks 14, 30, and 54; implies primary response]*: CRP of ≤3 mg/L and HBI score of ≤4 points, no ongoing steroid therapy, and no exit due to treatment failure.

Steroid use was defined as any systemic therapy, either oral or intravenous [including use of steroids for other conditions], but excluding single pre-infusion dosing with hydrocortisone.

PNR were required to exhibit primary non-response at week 14 and non-remission at week 54. PR were required to exhibit primary response or remission at week 14, and be in remission at week 54 [or week 30 if week 54 status was unknown]. Furthermore, within infliximab-treated patients, PNR and non-PNR were matched based on baseline immunomodulator use, baseline steroid use, age at first dose, baseline albumin, sex, and weight at study entry.

### 2.3. Whole blood RNA-seq

Whole blood was collected in RNA Tempus tubes [Applied Biosystems] and stored at −80°C until extraction [QIAsymphony PAXgene Blood RNA Kit, Qiagen]. RNA was quantified using the QuBit BR RNA [ThermoFisher], and RNA integrity was assessed with the 4200 TapeStation [Agilent]. RNA-seq libraries were prepared using the Kapa mRNA HyperPrep Kit, with depletion of rRNA and globin mRNA using the QIAseq FastSelect RNA Removal Kit, and adapter ligation with IDT xGEN Dual Index UMI adapters. A total of 1141 samples from 396 patients were sequenced. Raw sequencing data were demultiplexed with Picard^23^ and aligned to the reference genome [GRCh38] using STAR [v2.6.1d].^24^ Reads were deduplicated using UMI-tools^25^ and quantified against the Ensembl 96 gene annotation with featureCounts [v1.6.4].^26^

Outlier samples were excluded, defined as >2 standard deviations from the mean based on percentage of aligned reads in coding regions reported by Picard, percentage of unique reads, and number of unique reads. Samples with a sex mismatch against the documented sex were removed. As gene expression measured from bulk tissue is heavily dependent on cell composition,^27^ cell proportions of six common cell types in whole blood [CD4^+^ T cells, CD8^+^ T cells, B cells, NK cells, monocytes, and granulocytes] were estimated using the Houseman method^28^ from paired DNA methylation data.^29^ Samples missing clinical data and/or cell proportion estimates were removed. A total of 814 samples remained after filtering. To accommodate variability in sampling day, samples were mapped to timepoints based on Kennedy et al.’s^8^ windows around major visits: week 0 [week −4 to 0], week 14 [week 10–20], week 30 [week 22–38], and week 54 [week 42–66]. Samples taken at additional visits [LOR or exit] falling within one of the windows were mapped to that timepoint, unless the patient also had a major visit sample inside that window. The mapping of samples to timepoints is shown in Supplementary Figure S1a. The number of samples per patient ranged from one to four, with a median of three [Supplementary Figure S1b].

Counts were normalized for library size using edgeR [v3.28.1].^30^ Globin genes and short non-coding RNAs were removed. Genes with low expression were filtered, requiring genes to have at least 1.25 counts per million in >10% of samples and non-zero expression in >90% of samples. Expression data from 15 511 genes remained after filtering. Finally, log_2_ expression values were computed using variancePartition/voom.^31,32^

### 2.4. Statistical analyses

A full description of the statistical analyses can be found in the Supplementary Methods. In brief, DGE analyses were performed in R [v3.6.2],^33^ with the significance threshold set at a false discovery rate [FDR] of <0.05. Variance components analysis was used to identify influential variables for inclusion in DGE models [Supplementary Figure S2]. Cell proportion estimates were found to explain large fractions of expression variance, and adjusting for cell proportions reduced the number of significant associations but improved consistency between drug subgroups, with fewer highly significant modules showing significant drug-by-response interaction effects compared to the unadjusted models [Supplementary Figure S3]. As this study was not designed to compare between drug subgroups, we focused on models adjusted for cell composition, where the improved consistency allowed us to pool expression data from both subgroups for greater statistical power. For all DGE models, cell proportions, sequencing batch, age of onset [the patient’s age at disease diagnosis], disease duration, BMI, anti-TNF drug type, prior surgery, and smoking were included as fixed effects; and patient was included as a random effect.

Per-gene linear mixed-effects models fit using DREAM [variancePartition v1.16.1]^34^ were used to detect pairwise DGE between study groups. Additionally, natural cubic splines [splines::ns]^33^ were fit to explore non-linear expression trajectories over all four timepoints, modelling expression as a function of study day in each group [day 0 = day of first drug dose]. Different expression trajectories were detected by testing for differences in spline parameters between the PR and PNR groups. Significant genes from the spline analysis were hierarchically clustered by their mean expression in PR and PNR at each timepoint, and the gap statistic^35^ was used to define clusters of genes with distinct trajectories. The spline analyses were only performed with drug subgroups pooled, as relatively small sample sizes at weeks 30 and 54 precluded stratification by drug.

Rank-based gene set enrichment analyses [tmod::tmodCERNOtest, v0.46.2],^36^ using blood transcriptomic modules [BTMs] were performed to identify coordinately up- or downregulated gene sets. These modules represent sets of genes that are coexpressed in whole blood, derived by Li et al.^37^ [module names prefixed with ‘LI’] and Chaussabel et al.^38^ [prefixed ‘DC’] from publicly available expression datasets. Gene set overrepresentation analyses were run for BTMs [tmod::tmodHGtest] and other publicly available gene sets [gprofiler2::gost, v0.2.0].^39^

Single-sample gene set enrichment scores [ssGSEAs, https://github.com/broadinstitute/ssGSEA2.0/] were computed as a summary measure of module expression in a sample, both at baseline and at week 14. Predictive models using clinical variables, cell proportions, and module expression scores [baseline or week 14] to predict response status were constructed using caret [v6.0-86].^40^ Multiple predictive algorithms were evaluated: penalized and regularized logistic regression methods, parallel random forest, eXtreme Gradient Boosting, support vector machines with a radial basis, *k*-nearest neighbours, naive Bayes, and Gaussian process models. Bootstrapping [50 replicates] with the area under the curve [AUC] metric was used to tune models, evaluate internal performance, and perform model selection. Pairwise tests for the difference in AUCs were performed with pROC.^41^

### 2.5. Ethical statement

The South West Research Ethics committee approved the study [Research Ethics Committee reference: 12/SW/0323] in January 2013. Patients were included after providing informed, written consent. The study is registered with ClinicalTrials.gov identifier NCT03088449 and the protocol is available at https://www.ibdresearch.co.uk/pants/.

## 3. Results

### 3.1. Baseline module expression associated with post-induction primary non-response

**Figure 1.**
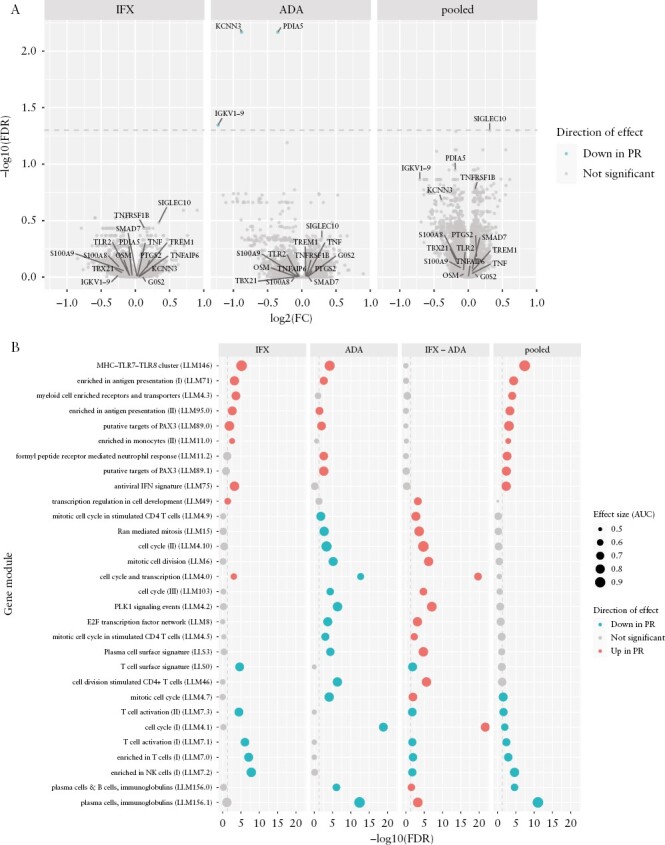
Baseline expression associated with primary response. [A] Volcano plots of differential gene expression between responders [PR] and non-responders [PNR] at week 0: for infliximab [IFX], adalimumab [ADA], or with drug subgroups pooled. Annotated genes show significant associations from this study and previously reported associations from the literature in both blood and gut biopsies. Dashed line shows significance threshold at FDR = 0.05. [B] Top gene modules differentially expressed between PR and PNR at week 0. Columns correspond to results for IFX, ADA, difference between IFX and ADA [IFX − ADA, i.e. the drug-by-response interaction], and pooled drug analyses. The top 30 modules ranked by minimum FDR in any column are shown. Dashed lines show significance thresholds at FDR = 0.05.

After RNA-seq quantification and quality control, expression data were available for 15 511 genes and 814 samples. These samples were from 324 patients, whose characteristics are shown in Table 1. We tested for associations between primary non-response and week 0 expression of genes [Figure 1a] and gene modules^37^ [Figure 1b], adjusting for cell composition and other influential covariates. Although no single gene was differentially expressed in the infliximab subgroup [86 PR, 59 PNR], expression of NK cell [LI.M7.2] and T cell [LI.M7.1, LI.M7.0] modules was significantly lower in responders. In the adalimumab subgroup [66 PR, 57 PNR], *PDIA5* (log_2_ fold change [FC] = −0.3512, FDR = 0.006777), *KCNN3* [log_2_ FC = −0.8798, FDR = 0.006777], and *IGKV1-9* [log_2_ FC = −1.223, FDR = 0.04518] expression was significantly lower in responders, accompanied by lower expression of plasma cell/immunoglobulin [LI.M156.0, LI.M156.1] and cell cycle [LI.M4.0, LI.M4.1] modules. This heterogeneity between drug subgroups was robust to model form [Figure S4] and differences in sample size between subgroups [Figure S5]. A pooled analysis was performed to identify modules consistently differentially expressed in both drug subgroups [152 PR, 116 PNR]. The MHC-TLR7-TLR8 cluster [LI.M146], antigen presentation [LI.M71, LI.M95.0], and myeloid cell enriched receptor and transporter [LI.M4.3] modules had higher expression at baseline in responders. Several of these module associations were largely or partially driven by major histocompatibility complex [MHC] class I and class II genes [Figure S6].

**Table 1.** Baseline patient characteristics for the PANTS RNA-seq subcohort.

	Adalimumab [ADA]	Infliximab [IFX]	Drugs pooled	*p*-value
Sex [Col %]				
Female	78 [48.4%]	89 [54.6%]	167 [51.5%]	
Male	83 [51.6%]	74 [45.4%]	157 [48.5%]	
Age of onset [years]				0.774
Mean [SD]	33.3 [15.4]	32.8 [15.3]	33.1 [15.3]	Wilcoxon rank-sum
Missing	0	0	0	
Disease duration [years]				0.546
Mean [SD]	6.1 [8.1]	5.9 [7.7]	6.0 [7.9]	Wilcoxon rank-sum
Missing	0	0	0	
Smoking status [Col %]				0.263
Current	28 [17.4%]	36 [22.1%]	64 [19.8%]	Fisher exact
Ex	55 [34.2%]	43 [26.4%]	98 [30.2%]	
Never	78 [48.4%]	84 [51.5%]	162 [50.0%]	
Crohn’s-related surgery [Col %]				0.549
No	114 [70.8%]	110 [67.5%]	224 [69.1%]	Fisher exact
Yes	47 [29.2%]	53 [32.5%]	100 [30.9%]	
On immunomodulator ever [Col %]				0.543
No	23 [14.3%]	28 [17.2%]	51 [15.7%]	Fisher exact
Yes	138 [85.7%]	135 [82.8%]	273 [84.3%]	
On immunomodulator at baseline [Col %]				0.912
No	79 [49.1%]	81 [49.7%]	160 [49.4%]	Fisher exact
Yes	82 [50.9%]	82 [50.3%]	164 [50.6%]	
On corticosteroids at baseline [Col %]				0.011
No	113 [70.2%]	92 [56.4%]	205 [63.3%]	Fisher exact
Yes	48 [29.8%]	71 [43.6%]	119 [36.7%]	
Baseline BMI				0.237
Mean [SD]	25.2 [6.2]	24.3 [5.5]	24.8 [5.9]	Wilcoxon rank-sum
Missing	0	0	0	
Primary response status [Col %]				0.263
Primary non-response	76 [47.2%]	66 [40.5%]	142 [43.8%]	Fisher exact
Primary response	85 [52.8%]	97 [59.5%]	182 [56.2%]	
CD8^+^ T cell [%]				0.380
Mean [SD]	2.8 [4.2]	2.8 [5.2]	2.8 [4.7]	Wilcoxon rank-sum
Missing	38	18	56	
CD4^+^ T cell [%]				0.752
Mean [SD]	9.2 [6.3]	9.2 [6.8]	9.2 [6.5]	Wilcoxon rank-sum
Missing	38	18	56	
B cell [%]				0.094
Mean [SD]	1.9 [2.0]	1.5 [1.9]	1.7 [1.9]	Wilcoxon rank-sum
Missing	38	18	56	
Monocyte [%]				0.497
Mean [SD]	8.9 [3.5]	9.2 [3.7]	9.0 [3.6]	Wilcoxon rank-sum
Missing	38	18	56	
NK cell [%]				0.683
Mean [SD]	1.9 [3.2]	1.9 [3.8]	1.9 [3.5]	Wilcoxon rank-sum
Missing	38	18	56	
Granulocyte [%]				0.911
Mean [SD]	74.3 [9.7]	74.3 [10.8]	74.3 [10.3]	Wilcoxon rank-sum
Missing	38	18	56	

Patient characteristics are stratified by drug subgroup. Values shown are count and percentage for categorical variables, and mean and standard deviation for continuous variables. Nominal *p*-values are reported for the comparison between drug subgroups.

In addition, we collated baseline markers of anti-TNF response in gut mucosal biopsies and blood from the literature.^14,15,18,42^ These were not significant in our per-gene DGE analyses [Figure 1a].

### 3.2. Expression changes from baseline to post-induction are largely amplified in primary responders

**Figure 2.**
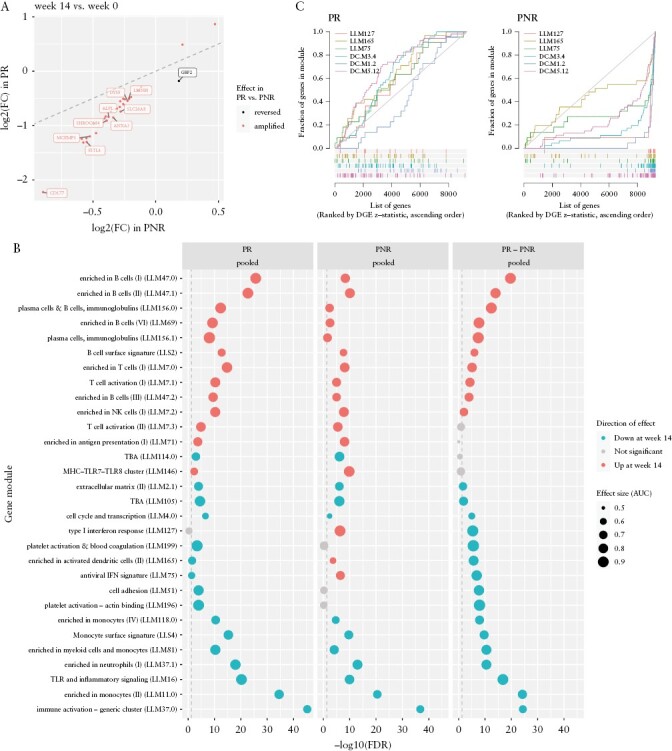
Expression changes from baseline to post-induction in responders and non-responders. [A] Expression log_2_ fold changes from week 0 to week 14 in primary responders [PR] and non-responders [PNR], for genes that were differentially expressed from week 0 to week 14 in both responders and non-responders, with a significantly different effect size between responders and non-responders [top ten labelled]. The identity line is shown by the dashed line. [B] Top modules differentially expressed between week 14 and week 0. Columns show effects in PR, PNR, and the PR minus PNR difference [the timepoint-by-response interaction]. The top 30 modules ranked by minimum FDR in any column are shown. Vertical dashed line shows significance threshold at FDR = 0.05. [C] Barcode plots showing interferon modules upregulated from week 0 to week 14 in PNR, but not in PR. Genes are ranked in ascending order by week 14 vs week 0 DGE *z*-statistic, with coloured bars indicating the rank of genes in a module. Curves show the cumulative fraction of genes in a module at a particular rank threshold. The area under the curve [AUC] reflects the effect size of the module association. Diagonal line shows the null of randomly distributed ranks. Modules sourced from Li et al.^37^ [prefixed ‘LI’] and Chaussabel et al.^38^ [prefixed ‘DC’].

To characterize the changes in gene expression induced by anti-TNF therapy, we compared expression at baseline to expression post-induction, and also estimated the difference between expression changes in responders and non-responders [the timepoint-by-response interaction]. As expression changes from week 0 to week 14 were relatively consistent between patients on infliximab and adalimumab after adjusting for cell composition [Figure S7], we pooled drug subgroups for these models. We found that 5572 and 626 genes were differentially expressed between week 14 and week 0 in responders and non-responders respectively, with 179 genes having a significant timepoint-by-response interaction. Of the genes differentially expressed in both responders and non-responders, and with a significant timepoint-by-response interaction, nearly all [31/32 genes] had an expression change that was amplified in responders [Figure 2a]. For example, *CD177*, a neutrophil marker upregulated during inflammation, was downregulated at week 14 in both responders [log_2_ FC = −2.225, FDR = 4.104 × 10^−17^] and non-responders [log_2_ FC = −0.8981, FDR = 0.004598], with significantly greater downregulation in responders [interaction FDR = 0.008247]. Modules differentially expressed between week 0 and week 14 included upregulation of B cell [LI.M47.0], plasma cell [LI.M156.0], and T cell activation [LI.M7.1] modules; and downregulation of immune activation [LI.M37.0], monocyte [LI.M11.0], neutrophil [LI.M37.1], and Toll-like receptor [TLR] and inflammatory signalling [LI.M16] modules [Figure 2b]. Statistically significant amplification of expression changes in responders was also observed at the module level, with nearly all module expression changes aligned in the same direction in responders and non-responders, but with larger effect sizes in responders.

In contrast, *GBP2* [a member of a family of guanylate-binding proteins induced by interferons^43^] was downregulated from week 0 to week 14 in responders [log_2_ FC = −0.1783, FDR = 0.004878], but upregulated in non-responders [log_2_ FC = 0.1849, FDR = 0.04502; interaction FDR = 0.005977]. At the module level, upregulation of the type I interferon response [LI.M127], activated dendritic cell [LI.M165], and antiviral IFN signature [LI.M75] modules was observed in non-responders but not in responders [Figure 2b]. Extended gene set enrichment analyses including additional modules from Chaussabel et al.^38^ also identified interferon modules significantly upregulated at week 14 in non-responders: DC.M3.4, containing *STAT2*, *GBP5*, and *PARP14* [FDR = 3.447 × 10^−21^]; and two modules containing *IFIT3* and *GBP2*, DC.M1.2 [FDR = 9.492 × 10^−16^] and DC.M5.12 [FDR = 1.355 × 10^−13^]. None of these modules were differentially expressed from week 0 to week 14 in responders [Figure 2c], suggesting upregulation of interferon pathways post-induction occurs uniquely in primary non-responders.

### 3.3. Sustained expression differences between responders and non-responders during maintenance

**Figure 3.**
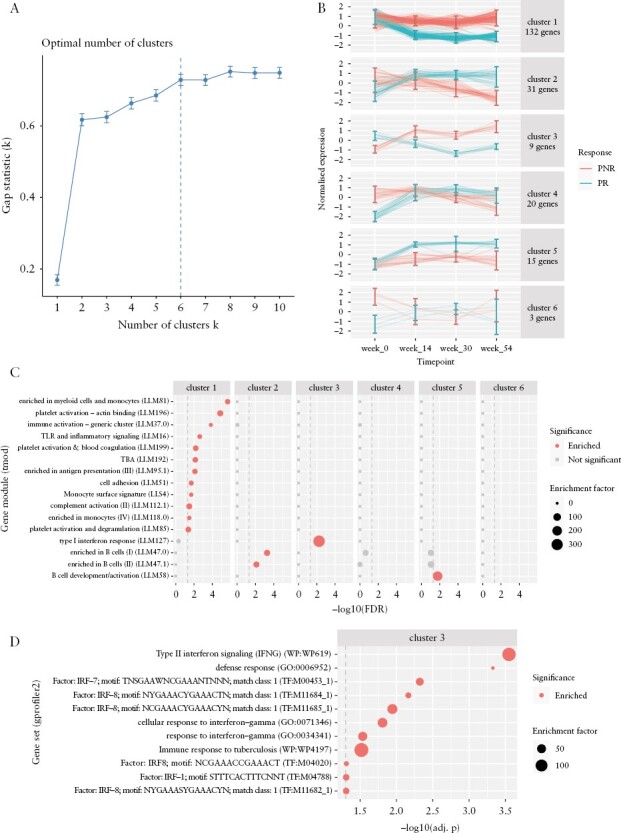
Expression differences between responders [PR] and non-responders [PNR] during maintenance. [A] Gap statistic vs cluster number *k* from hierarchical clustering of genes with significant expression differences between PR and PNR over all timepoints. Error bars derived from 500 bootstraps. The optimal cluster number was selected to be *k* = 6 by the factoextra::fviz_nbclust firstSEmax criteria [https://rpkgs.datanovia.com/factoextra/index.html]. [B] Normalized expression over study timepoints for genes in each cluster; 95% confidence intervals for expression are shown for each group at each timepoint. [C] Gene modules enriched in each cluster from gene set overrepresentation analyses. Modules significantly enriched in any cluster are shown. Vertical dashed line shows significance threshold at FDR = 0.05. [D] Gene sets enriched in cluster 3 from gene set overrepresentation analyses using gprofiler2::gost.^39^ Vertical dashed line shows significance threshold at an adjusted *p*-value = 0.05 [gost g:SCS multiple testing correction method].

As PANTS was an observational study, it was possible to retain patients who continued with anti-TNF therapy even after meeting the study definition of PNR at week 14, enabling us to sample the blood transcriptome at weeks 30 and 54 during the maintenance period. Utilizing all 814 samples over the four study timepoints, we tested for general differences in expression trajectory over time, detecting 210 differentially expressed genes between responders and non-responders after adjustment for cell composition. To visualize the expression of these genes and identify common patterns of expression change during anti-TNF therapy, significant genes were hierarchically clustered by their expression. Six clusters were identified [Figure 3a], each with distinct expression trajectories for responders and non-responders [Figure 3b]. Cluster 1 largely comprised genes previously found to have a significant difference in expression change from week 0 to week 14 between responders and non-responders [97/132 genes in the cluster]. The most significant gene was *KREMEN1* [FDR = 4.287 × 10^−4^], part of an inflammatory apoptotic pathway in the gut epithelium.^44^ Cluster 1 genes were enriched in modules associated with myeloid cells and monocytes [LI.M81, hypergeometric test, FDR = 2.115 × 10^−6^], platelet activation [LI.M196, 1.348 × 10^−5^], immune activation [LI.M37.0, 1.436 × 10^−4^], and TLR and inflammatory signalling [LI.M16, FDR = 2.365 × 10^−3^] [Figure 3c]. Expression trajectories showed cluster 1 genes were more downregulated from baseline in responders than in non-responders, probably representing a lower inflammatory state in responders by week 14 that is sustained at weeks 30 and 54. An opposing trend was observed in cluster 5, which contained genes enriched for B cell development/activation [LI.M58, FDR = 0.01653] that were more upregulated from baseline in responders than non-responders.

Cluster 3 was uniquely enriched for the type I interferon response [LI.M127, FDR = 0.005681] [Figure 3c]. Subsequent enrichment analyses using publicly available gene sets^39^ revealed enrichments for type II interferon signalling [WP:WP619, adj. *p* = 2.826 × 10^−4^], and for genes containing putative transcription factor [TF] binding motifs for interferon regulatory factors IRF7 [TF:M00453_1, adj. *p* = 0.004768] and IRF8 [TF:M11684_1, adj. *p* = 0.006853; TF:M11685_1, adj. *p* = 0.01136] [Figure 3d]. The genes in cluster 3 showed opposing directions of expression change from week 0 to week 14 in responders vs non-responders, generating expression differences at week 14 that were also sustained at weeks 30 and 54. A significant interaction between week 0 to week 14 expression change and response status from the per-gene differential expression analyses was observed for eight of the nine genes in the cluster [*STAT1*, *BATF2*, *GBP1*, *GBP5*, *IRF1*, *TAP1*, *APOL1*, *APOL2*], many of which are key interferon signalling genes.^43,45,46^ Unlike the majority of genes that followed trajectories of greater expression change in responders, genes in interferon response pathways were uniquely upregulated in non-responders after anti-TNF therapy.

### 3.4. Prediction of primary non-response from gene expression and clinical variables

**Figure 4.**
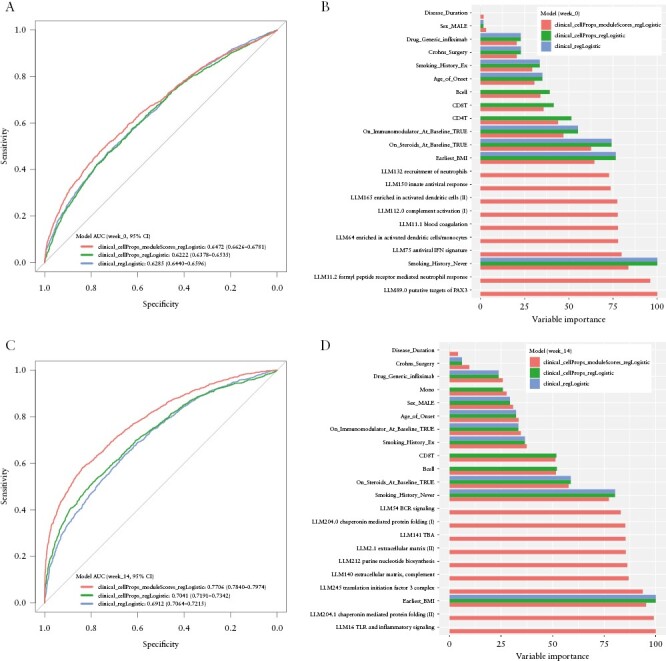
Prediction of response status from clinical variables, cell proportions, and expression data. Receiver operating characteristic [ROC] curves for the caret regLogistic method^40^ trained on each predictor dataset are shown at baseline [A] and week 14 [C]. ROC curves were plotted after merging all 50 resamples. Primary non-response was used as the positive class. DeLong 95% confidence intervals for the AUC are shown. The ten most important variables from models trained on each predictor dataset are shown for baseline [B] and week 14 [D] models. The overall variable importance score is computed from the absolute value of the *t*-statistic for each predictor from the final tuned models. Missing bars denote variables that were not in the predictor dataset for that model.

Given there were numerous module-level associations with primary response status, we evaluated the ability to predict response from module expression. For predictive models using only baseline data, the median resampling AUCs over all combinations of algorithms and predictor sets evaluated ranged from 0.5541 to 0.6686 [Supplementary Figure S8]. The best-performing model was regularized logistic regression [caret regLogistic model^40^; hyperparameters: cost = 0.25, loss = L1, epsilon = 0.01] using clinical variables, cell proportions, and module scores from ssGSEA as predictors, giving a median resampling AUC of 0.6686, a median sensitivity of 0.5392, and a median specificity of 0.6852, where non-response was the positive class, with a prevalence of 43% [116/268]. Including cell proportions and module scores did improve predictive performance compared to using only clinical variables [bootstrap *p* = 0.02629], but the increase in AUC was only 2.5% [Figure 4a]. This suggests that clinical variables provided the greatest contributions to baseline prediction, especially those variables with consistently high importance scores [high absolute *t*-statistics]: smoking history, BMI, and baseline steroid usage [Figure 4b].

Model performance was improved by utilizing week 14 cell proportions and module scores instead of baseline [median resampling AUC range 0.6216–0.7957] [Supplementary Figure S9]. The prevalence of non-response at week 14 was 42% [104/246]. Again, the best performing model was regularized logistic regression incorporating clinical variables, cell proportions, and module scores as predictors [median resampling AUC = 0.7957, sensitivity = 0.6248, specificity = 0.7961]. Adding week 14 module scores to the predictor dataset had a larger benefit, with a 6.5% increase in AUC comparing the full model to the model including only clinical variables and cell proportions [bootstrap *p* = 2.306 × 10^−10^] [Figure 4c]. The modules with the highest variable importance included TLR and inflammatory signalling [LI.M16], chaperonin-mediated protein folding [LI.M204.0, LI.M204.1], and translation initiation factor 3 complex [LI.M245] modules [Figure 4d]. Greater predictive performance at week 14 than baseline probably reflects the larger expression differences observed between responders and non-responders after the induction period.

## 4. Discussion

We found substantial differences in whole blood gene expression between anti-TNF primary responders and non-responders in the PANTS cohort. At baseline, three single-gene associations detected in the adalimumab subgroup implicated similar cell types; *IGKV1-9* encodes the immunoglobulin light chain variable region that forms part of antibodies produced by plasma cells, *KCNN3* is annotated to a plasma cell surface signature module from Li et al.^37^ [LI.S3], and the expression of both *KCNN3* and *PDIA5* is high in plasma cells [www.proteinatlas.org/humanproteome/immune+cell, v21.1] and positively correlated with plasmablast frequencies in blood.^47^ These genes were downregulated in responders, as was the expression of plasma cell and immunoglobulin modules. In keeping with our observations, Martin et al.^17^ identified plasma cells as part of a correlated module of cell populations, where lower module expression in gut biopsies was associated with better response to anti-TNFs. Baseline plasma cell abundances in gut biopsies have also been reported to be lower in responders, albeit in relatively small cohorts of infliximab patients.^16^ Our findings lend credence that associations driven by immune cells observed in gut biopsies may also be observable in blood, a more accessible tissue.

Previously reported single-gene baseline markers in gut biopsies and blood were non-significant in this study. For example, *TREM1*^16,18^ was not significantly differentially expressed between responders and non-responders in blood samples from PANTS patients. Our observation is consistent with two recent trials of comparable sample size, SERENE-CD and SERENE-UC, where baseline blood *TREM1* expression was not predictive of response in either CD or UC patients.^48^ A variety of factors could explain failures to replicate reported markers from study to study. Many existing studies pool heterogeneous cohorts of patients taking different anti-TNF drugs due to the scarcity of large datasets, but even between arms of the PANTS study, we observed within-study differences in expression. Additional between-study variation can arise from differences in clinical setting, tissues sampled [e.g. blood vs gut biopsies], and definition of primary response [e.g. endoscopic vs clinical parameters]. Any two studies are unlikely to have adjusted for the same combinations of covariates in modelling, including covariates such as cell composition that heavily influence bulk expression data. Finally, small sample sizes have considerable sampling error. We recommend the use of set-based methods over single-gene association tests for identification of biomarkers, as drawing on differences in multiple genes improves statistical power, and may also improve reproducibility between studies. Despite the small number of single-gene associations in PANTS, we detected module-level associations that were consistent between drug subgroups, revealing a higher baseline expression of MHC and antigen presentation modules in primary responders. Genetic variation in the MHC region has been previously linked to immunogenicity rates in PANTS, where carriage of HLA-DQA1*05 was associated with a 2-fold increase in the risk for developing anti-drug antibodies, although the mechanism is yet unknown.^22^

Utilizing the longitudinal design of PANTS, we characterized changes in blood gene expression post-induction. Reduced expression of immune activation, monocyte, and neutrophil modules in responders at week 14 is consistent with successful drug inhibition of TNF-mediated inflammation, which correlates with reduced neutrophil activation and reduced monocyte recruitment.^49^ Apoptosis of monocytes induced by anti-TNF in CD patients has also been previously described.^44^ Certain B cell subsets are reduced in the blood of IBD patients compared to controls,^50^ so upregulation of B cell modules at week 14 may also represent a shift towards health. Similar expression changes were observed in responders and non-responders, but with greater magnitude in responders, potentially suggesting a continuum of response. Gaujoux et al.^16^ found that changes in cell proportions after anti-TNF treatment were amplified in responders; here we demonstrate a similar trend at the transcriptional level in PANTS. Post-induction expression differences between responders and non-responders were sustained at weeks 30 and 54 during the anti-TNF maintenance period. Kennedy et al.^8^ found that ‘continuing standard dosing regimens after primary non-response was rarely helpful’ for inducing remission by week 54. This phenomenon may also be reflected in the blood transcriptome, although non-responders in this study were selected to exclude patients in remission by week 54, so expression trajectories for non-responders at week 14 who eventually achieved remission could not be observed, and differences in trajectory between PANTS responders and non-responders may be exaggerated.

Unlike the majority of baseline vs post-induction associations, expression changes in genes and modules in the interferon pathway were uniquely upregulated in PANTS non-responders. Previous studies in IBD are conflicting, with Samie et al.^51^ reporting elevated expression of interferon pathway genes in colonic biopsies from non-responders compared to responders, with no significant change pre- vs post-treatment [*n* ≈ 40]; and Mavragani et al.^20^ reporting a post-treatment reduction in blood interferon expression only in non-responders [*n* = 30]. In studies of rheumatoid arthritis [RA], another IMID with licensed anti-TNF therapies, increases in type I interferon-regulated gene expression in blood after infliximab treatment were associated with poor clinical response [discovery *n* = 15, validation *n* = 18].^52^ A systematic review of our study with other studies reporting similar associations between interferon pathway genes and anti-TNF response would not only help resolve the direction of effect, if any, but also provide an opportunity to consider the shared biology of anti-TNF response in IBD, RA, and other IMIDs.

We were unable to build clinically useful predictive models of response incorporating expression data. Using only baseline clinical variables, Kennedy et al.^8^ used logistic regression with stepwise variable selection based on Akaike’s information criterion [AIC] to predict response in the full PANTS cohort, achieving AUCs of 0.53 (95% confidence interval [CI] 0.46–0.59) for infliximab patients and 0.54 [0.46–0.62] for adalimumab patients. Whilst our best-performing baseline model achieved a 13% improvement in AUC, expression data only contributed a small amount of predictive power on top of clinical variables and cell composition. Unsurprisingly, models had greater predictive power when provided with week 14 expression and cell composition data, and adding expression data also provided a comparatively large increase in AUC. This suggests that when expression differences between responders and non-responders are sufficiently large, transcriptomic markers do provide unique information, and are not simply proxies for clinical variables or coarse estimates of blood cell composition. A potential route to more effective prediction is to consider whether expression differences arising early in the induction period can discriminate between responders and non-responders. For example, Mesko et al.^53^ found that week 2 blood gene expression was predictive of infliximab response in CD [discovery *n* = 20, validation *n* = 20] and RA [discovery *n* = 19, validation *n* = 15] patients. More recently, Mishra et al.^54^ trained random forest models using blood DNA methylation and gene expression measured in IBD patients receiving infliximab [*n* = 37]. They did not find consistent baseline-only predictive signatures, but a model combining baseline with week 2 measurements predicted response in the Mesko et al. cohort with 85% accuracy [95% CI: 62–97%]. As we observed in PANTS, expression differences between responders and non-responders were far greater by week 14 than at baseline. Post-induction associations were also more consistent between drug subgroups, as baseline differences are diluted by the large transcriptomic perturbation from taking an anti-TNF. Expression changes in the innate immune system are observable within hours of treatment initiation,^54^ and robust prediction of non-response within that timeframe may be more valuable than a less reliable prediction at baseline.

An important limitation of our analyses is that PANTS was not designed to directly compare between drug subgroups. Differences between patient groups taking different anti-TNF drugs can arise from patient and physician preferences, influenced by cost, disease severity, location, and comorbidities. Unsurprisingly, many associations with response had significantly different effect sizes in the infliximab and adalimumab patient subgroups. We found that adjusting DGE models for estimated proportions of major cell types as a proxy for these uncontrolled factors alleviated heterogeneity between subgroups. However, the adjustment is unlikely to work well for rare cell types, and thus the associations we report may reflect differences in cell proportions rather than per-cell expression. Given the myriad of factors that could drive the observed heterogeneity, we strongly caution against interpreting associations with different effects in the PANTS infliximab and adalimumab subgroups as drug-driven differences with biological significance, and recommend that future transcriptomic studies consider influential factors such as cell composition. Additionally, PANTS defined response pragmatically as a composite, clinical outcome incorporating physician evaluation, disease severity scores, and serum CRP. Although this clinical outcome is significantly associated with faecal calprotectin, which correlates with endoscopic outcomes,^8^ our results would have been strengthened if paired endoscopic data were available.

In conclusion, we observed significant differences in gene module expression between responders and non-responders to anti-TNF therapy in the whole blood of PANTS CD patients at baseline and post-treatment timepoints. Interferon-induced genes were uniquely upregulated post-induction in non-responders, going against the general trend of amplified transcriptomic change in responders vs non-responders. We were unable to robustly predict response from baseline data with our current sample size. To obtain more accurate predictions, utilizing large upcoming datasets with paired drug response phenotypes and transcriptomic data such as the 1000IBD project will be essential.^55^

## Supplementary Material

jjad166_suppl_Supplementary_Figure_S1

jjad166_suppl_Supplementary_Figure_S2

jjad166_suppl_Supplementary_Figure_S3

jjad166_suppl_Supplementary_Figure_S4

jjad166_suppl_Supplementary_Figure_S5

jjad166_suppl_Supplementary_Figure_S6

jjad166_suppl_Supplementary_Figure_S7

jjad166_suppl_Supplementary_Figure_S8

jjad166_suppl_Supplementary_Figure_S9

jjad166_suppl_Supplementary_Figure_S10

jjad166_suppl_Supplementary_Figure_S11

jjad166_suppl_Supplementary_Data

jjad166_suppl_Supplementary_Material

## Data Availability

Pre-processed gene expression count data and anonymized patient metadata underlying the DGE analyses, as well as other summary-level data associated with the Figures in this article, can be found in the online Supplementary Data files. Individual participant de-identified raw data that underlie the results reported in this article will be available immediately after publication for a period of 5 years. The data will be made available to investigators whose proposed use of the data has been approved by an independent review committee. Analyses will be restricted to the aims in the approved proposal. Proposals should be directed to Tariq Ahmad [tariq.ahmad1@nhs.net]. To gain access data requestors will need to sign a data access agreement.

## UK Inflammatory Bowel Disease Pharmacogenetics Study Group

Dr Vinod Patel^a^, Dr Zia Mazhar^b^, Dr Rebecca Saich^c^, Dr Ben Colleypriest^d^, Dr Tony C Tham^e^, Dr Tariq H Iqbal^f^, Dr Vishal Kaushik^g^, Dr Senthil Murugesan^h^, Dr Salil Singhi, Dr Sean Weaver^j^, Dr Cathryn Preston^k^, Dr Assad Butt^l^, Dr Melissa Smith^l^, Dr Dharamveer Basude^m^, Dr Amanda Beale^m^, Dr Sarah Langlands^n^, Dr Natalie Direkze^n^, Dr Miles Parkes^o^, Dr Franco Torrente^o^, Dr Juan De La Revella Negro^o^, Dr Chris Ewen MacDonald^p^, Dr Stephen M Evans^q^, Dr Anton V J Gunasekera^r^, Dr Alka Thakur^q^, Dr David Elphick^s^, Dr Achuth Shenoy^t^, Prof Chuka U Nwokolo^u^, Dr Anjan Dhar^v^, Dr Andrew T Cole^w^, Dr Anurag Agrawal^x^, Dr Stephen Bridger^y^, Dr Julie Doherty^z^, Dr Sheldon C Cooper^aa^, Dr Shanika de Silva^aa^, Dr Craig Mowat^cc^, Dr Phillip Mayhead^dd^, Dr Charlie Lees^ee^, Dr Gareth Jones^ee^, Dr Tariq Ahmad^ff^, Dr James W Hart^ff^, Dr Daniel R Gaya^gg^, Prof Richard K Russell^hh^, Dr Lisa Gervais^hh^, Dr Paul Dunckley^ii^, Dr Tariq Mahmood^jj^, Dr Paul J R Banim^kk^, Dr Sunil Sonwalkar^ll^, Dr Deb Ghosh^mm^, Dr Rosemary H Phillips^mm^, Dr Amer Azaz^nn^, Dr Shaji Sebastian^nn^, Dr Richard Shenderey^oo^, Dr Lawrence Armstrong^pp^, Dr Claire Bell^pp^, Dr Radhakrishnan Hariraj^qq^, Dr Helen Matthews^rr^, Dr Hasnain Jafferbhoy^ss^, Dr Christian P Selinger^tt^, Dr Veena Zamvar^tt^, Prof John S De Caestecker^uu^, Dr Anne Willmott^uu^, Mr Richard Miller^vv^, Dr Palani Sathish Babu^ww^, Dr Christos Tzivinikos^xx^, Dr Stuart L Bloom^yy^, Dr Guy Chung-Faye^zz^, Prof Nicholas M Croft^aaa^, Dr John ME Fell^bbb^, Dr Marcus Harbord^ccc^, Dr Ailsa Hart^ddd^, Dr Ben Hope^zz^, Dr Peter M Irving^eee^, Prof James O Lindsay^fff^, Dr Joel E Mawdsley^ggg^, Dr Alistair McNair^hhh^, Dr Kevin J Monahan^ccc^, Dr Charles D Murray^iii^, Prof Timothy Orchard^jjj^, Dr Thankam Paul^kkk^, Dr Richard Pollok^kkk^, Dr Neil Shah^lll^, Dr Sonia Bouri^ddd^, Dr Matt W Johnson^mmm^, Dr Anita Modi^nnn^, Dr Kasamu Dawa Kabiru^ooo^, Dr B K Baburajan^ppp^, Prof Bim Bhaduri^ppp^, Dr Andrew Adebayo Fagbemi^qqq^, Dr Scott Levison^rrr^, Dr Jimmy K Limdi^sss^, Dr Gill Watts^ttt^, Dr Stephen Foley^uuu^, Dr Arvind Ramadas^vvv^, Dr George MacFaul^www^, Dr John Mansfield^xxx^, Dr Leonie Grellier^yyy^, Dr Mary-Anne Morris^zzz^, Dr Mark Tremelling^zzz^, Prof Chris Hawkey^aaaa^, Dr Sian Kirkham^aaaa^, Dr Charles PJ Charlton^aaaa^, Dr Astor Rodrigues^bbbb^, Prof Alison Simmons^cccc^, Dr Stephen J Lewis^dddd^, Dr Jonathon Snook^eeee^, Dr Mark Tighe^eeee^, Dr Patrick M Goggin^ffff^, Dr Aminda N De Silva^gggg^, Prof Simon Lal^hhhh^, Dr Mark S Smith^iiii^, Dr Simon Panter^jjjj^, Dr Fraser Cummings^kkkk^, Dr Suranga Dharmisari^kkkk^, Dr Martyn Carter^llll^, Dr David Watts^mmmm^, Dr Zahid Mahmood^nnnn^, Dr Bruce McLain^oooo^, Dr Sandip Sen^pppp^, Dr Anna J Pigott^qqqq^, Dr David Hobday^rrrr^, Dr Emma Wesley^ssss^, Dr Richard Johnston^tttt^, Dr Cathryn Edwards^tttt^, Dr John Beckly^uuuu^, Dr Deven Vani^vvvv^, Dr Subramaniam Ramakrishnan^wwww^, Dr Rakesh Chaudhary^xxxx^, Dr Nigel J Trudgill^yyyy^, Dr Rachel Cooney^yyyy^, Dr Andy Bell^zzzz^, Dr Neeraj Prasad^aaaaa^, Dr John N Gordon^bbbbb^, Prof Matthew J Brookes^ccccc^, Dr Andy Li^ddddd^, Dr Stephen Gore^eeeee^

^a^Tameside Hospital NHS Foundation Trust, Ashton U Lyne

^b^Basildon and Thurrock University Hospitals NHS Foundation Trust, Basildon

^c^Hampshire Hospitals NHS Foundation Trust, Basingstoke

^d^Royal United Hospital, Bath

^e^Ulster Hospital, Belfast

^f^University Hospital's Birmingham NHS Foundation Trust, Birmingham

^g^East Lancashire NHS Teaching Trust, Blackburn

^h^Blackpool Teaching Hospitals NHS Foundation Trust, Blackpool

^i^Bolton NHS Trust, Bolton

^j^Royal Bournemouth Hospital, Bournemouth

^k^Bradford Teaching Hospitals Foundation Trust - (St Lukes Hospital &Bradford Royal Infirmary), Bradford

^l^Brighton and Sussex University Hospitals NHS Trust, Brighton

^m^University Hospitals Bristol NHS Foundation Trust, Bristol

^n^Frimley Park Hospital NHS Foundation Trust, Camberley

^o^Cambridge University Hospitals NHS Foundation Trust, Cambridge

^p^North Cumbria University Hospitals NHS Trust, Carlisle

^q^Ashford & St Peter's Hospitals NHS Foundation Trust, Chertsey

^r^St Peter's Hospital, Chertsey

^s^Chesterfield Royal NHS Foundation Trust, Chesterfield

^t^Colchester Hospital University NHS Foundation Trust, Colchester

^u^University Hospitals Coventry and Warwickshire NHS Trust, Coventry

^v^County Durham and Darlington NHS Foundation Trust, Darlington

^w^Derby Hospital NHS Foundation NHS Trust, Derby

^x^Doncaster and Bassetlaw Hospitals NHS Foundation Trust, Doncaster

^y^Dorset County Hospital NHS Foundation Trust, Dorchester

^z^Dorset County Hospitals Foundation Trust, Dorchester

^aa^Dudley Group NHS Foundation Trust, Dudley

^bb^Russells Hall Hospital, The Dudley Group NHS Foundation Trust, Dudley

^cc^Ninewells Hospital & Medical School, Dundee

^dd^East Sussex Healthcare Trust, Eastborne

^ee^NHS Lothian, Edinburgh

^ff^Royal Devon and Exeter NHS Foundation Trust, Exeter

^gg^Glasgow Royal Infirmary, Glasgow

^hh^Royal Hospital for Children, Glasgow

^ii^Gloucestershire Hospitals NHS Trust, Gloucester

^jj^United Lincolnshire Hospitals NHS Trust, Grantham

^kk^James Paget University Hospitals NHS Foundation Trust, Great Yarmouth

^ll^Calderdale and Huddersfield NHS Trust, Halifax

^mm^Princess Alexandra Hospital NHS Trust, Harlow

^nn^Hull and East Yorkshire NHS Trust, Hull

^oo^Airedale NHS Foundation Trust, Keighley

^pp^Crosshouse Hospital, Kilmarnock

^qq^The Queen Elizabeth Hospital NHS Foundation Trust, Kings Lynn

^rr^Kingston Hospital NHS Trust, Kingston upon Thames

^ss^NHS Fife, Kirkcaldy

^tt^Leeds Teaching Hospitals NHS Trust, Leeds

^uu^University Hospitals of Leicester NHS Trust, Leicester

^vv^Mid Cheshire Hospitals NHS Foundation Trust, Leighton

^ww^United Lincolnshire Hospitals NHS Trust, Lincoln

^xx^Alder Hey Childrens Hospital, Liverpool

^yy^University College London Hospitals NHS Foundation Trust, London

^zz^Kings College Hospital NHS Foundation Trust, London

^aaa^Royal London Childrens Hospital, Barts Health NHS Trust, London

^bbb^Chelsea & Westminster Hospital, London

^ccc^Chelsea and Westminster Hospital NHS Foundation, London

^ddd^North West London Hospitals NHS Trust, London

^eee^Guys & St Thomas' NHS Foundation Trust, London

^fff^Barts and The London NHS Trust, London

^ggg^Guy's and St Thomas' NHS trust, London

^hhh^Lewisham and Greenwich Healthcare NHS Trust, London

^iii^Royal Free London NHS Foundation Trust, London

^jjj^Imperial College Healthcare NHS Trust, London

^kkk^St George's Healthcare NHS Trust, London

^lll^Great Ormond Street Hospital for Children NHS Foundation Trust, London

^mmm^The Luton & Dunstable University Hospital, Luton

^nnn^Luton and Dunstable Hospital Foundation Trust, Luton

^ooo^The Luton & Dunstable University Hospital, Luton

^ppp^Maidstone and Tunbridge Wells NHS Trust, Maidstone

^qqq^Manchester University Hospitals NHS Foundation Trust, Manchester

^rrr^Central Manchester University Hospitals NHS Foundation Trust, Manchester

^sss^The Pennine Acute Hospitals NHS Trust, Manchester

^ttt^Manchester University NHS Foundation Trust, Wythenshawe Hospital, Manchester

^uuu^Sherwood Forest Hospitals NHS Foundation Trust, Mansfield

^vvv^South Tees Hospital NHS Foundation Trust, Middlesbrough

^www^Milton Keynes Hospital NHS Foundation Trust, Milton Keynes

^xxx^Newcastle Upon Tyne Hospital Trust, Newcastle

^yyy^Isle of Wight NHS Foundation Trust, Newport

^zzz^Norfolk & Norwich University Hospital NHS Foundation Trust, Norwich

^aaaa^Nottingham University Hospitals NHS Trust, Nottingham

^bbbb^Oxford University Hospitals NHS Foundation Trust, Oxford

^cccc^Oxford University Hospitals NHS Trust, Oxford

^dddd^Plymouth Hospitals NHS Trust, Plymouth

^eeee^Poole Hospital NHS Foundation Trust, Poole

^ffff^Portsmouth Hospitals NHS Trust, Portsmouth

^gggg^Royal Berkshire NHS Foundation Trust, Reading

^hhhh^Salford Royal NHS Foundation Trust, Salford

^iiii^Shrewsbury and Telford Hospital NHS Trust, Shrewsbury

^jjjj^South Tyneside NHS Foundation Trust, South Shields

^kkkkk^Southampton University Hospitals NHS Trust, Southampton

^llll^East and North Herts NHS Trust, Stevenage

^mmmm^NHS Forth Valley, Stirling

^nnnn^Stockport NHS foundation Trust, Stockport

^oooo^North Tees and Hartlepool NHS Foundation Trust, Stockton

^pppp^University Hospitals of North Staffordshire, Stoke-on Trent

^qqqq^University Hospitals of North Midlands NHS Trust, Stoke-on Trent

^rrrr^City Hospitals Sunderland NHS Foundation Trust, Sunderland

^ssss^Taunton and Somerset NHS Foundation Trust, Taunton

^tttt^South Devon Healthcare NHS Foundation Trust, Torquay

^uuuu^Royal Cornwall Hospitals NHS Trust, Truro

^vvvv^Mid Yorkshire Hospitals NHS Trust, Wakefield

^wwww^Warrington& Halton NHS Foundation, Warrington

^xxxx^West Hertfordshire Hospitals NHS Trust, Watford

^yyyy^Sandwell and West Birmingham Hospitals NHS Trust, West Bromwich

^zzzz^Weston Area Health NHS Trust, Weston-Super-Mare

^aaaaa^Royal Albert Edward Infirmary, Wrightington, Wigan & Leigh NHS Foundation Trust, Wigan

^bbbbb^Hampshire Hospitals NHS Foundation Trust, Winchester

^ccccc^Royal Wolverhampton Hospitals NHS Trust, Wolverhampton

^ddddd^Western Sussex Hospitals NHS Trust, Worthing

^eeeee^Yeovil District Hospital NHS Foundation Trust, Yeovil
