# Supplementary figures and images for: Baseline Expression of Immune Gene Modules in Blood is Associated With Primary Response to Anti-TNF Therapy in Crohn’s Disease Patients
Source: J Crohns Colitis. 2023 Sep 30;18(3):431–45. doi: 10.1093/ecco-jcc/jjad166 (PMC10906954; doi:10.1093/ecco-jcc/jjad166)

a

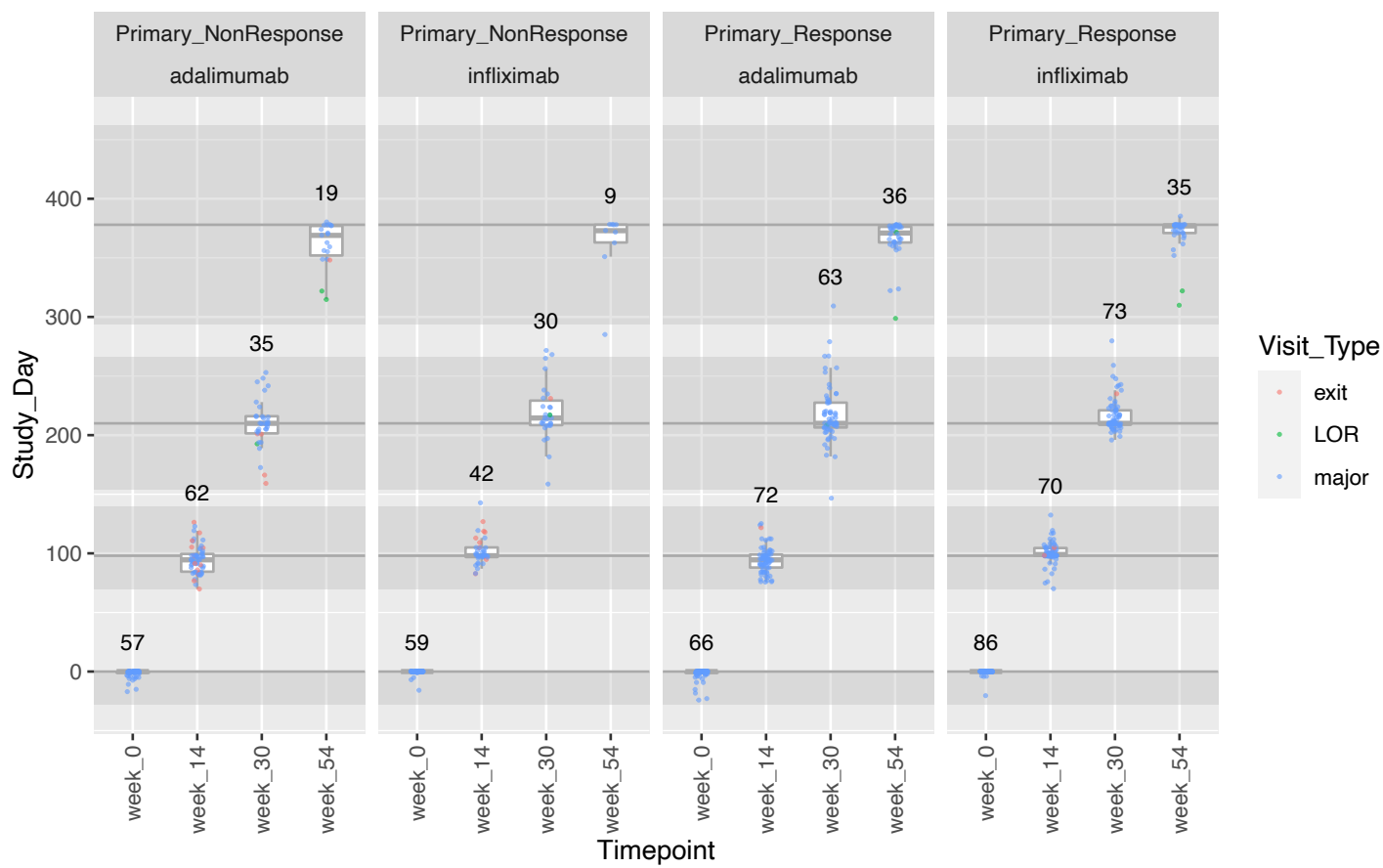

b

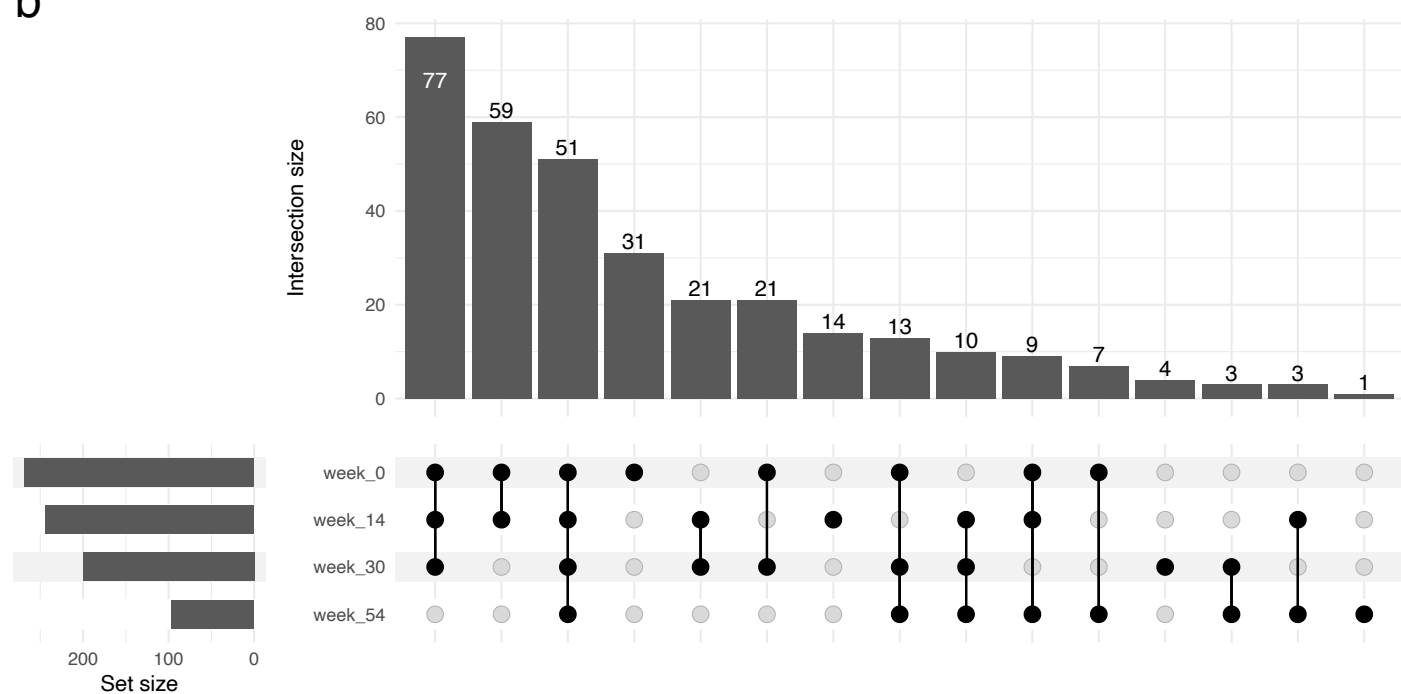

Supplement: jjad166_suppl_Supplementary_Figure_S1 [file jjad166_suppl_supplementary_figure_s1.pdf]

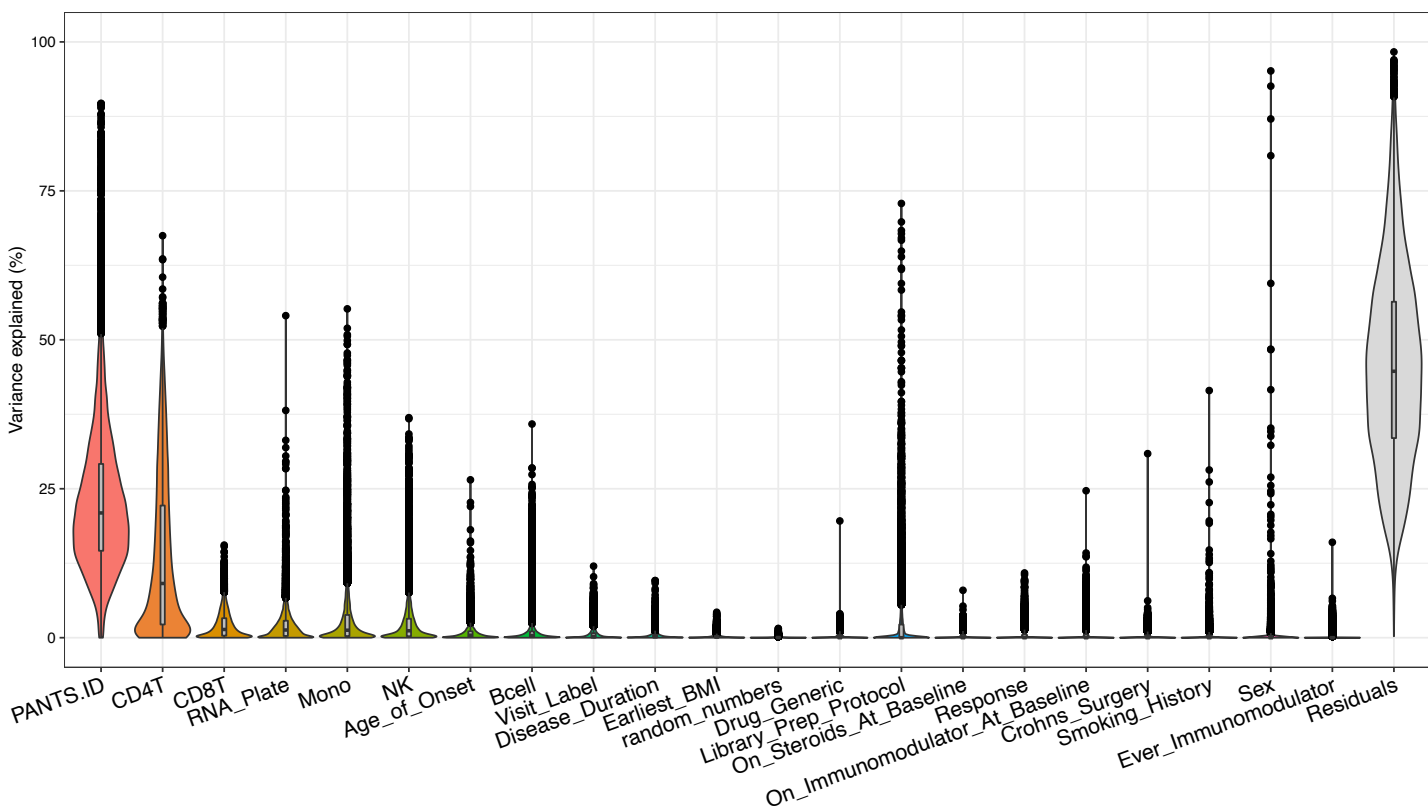

Supplement: jjad166_suppl_Supplementary_Figure_S2 [file jjad166_suppl_supplementary_figure_s2.pdf]

a

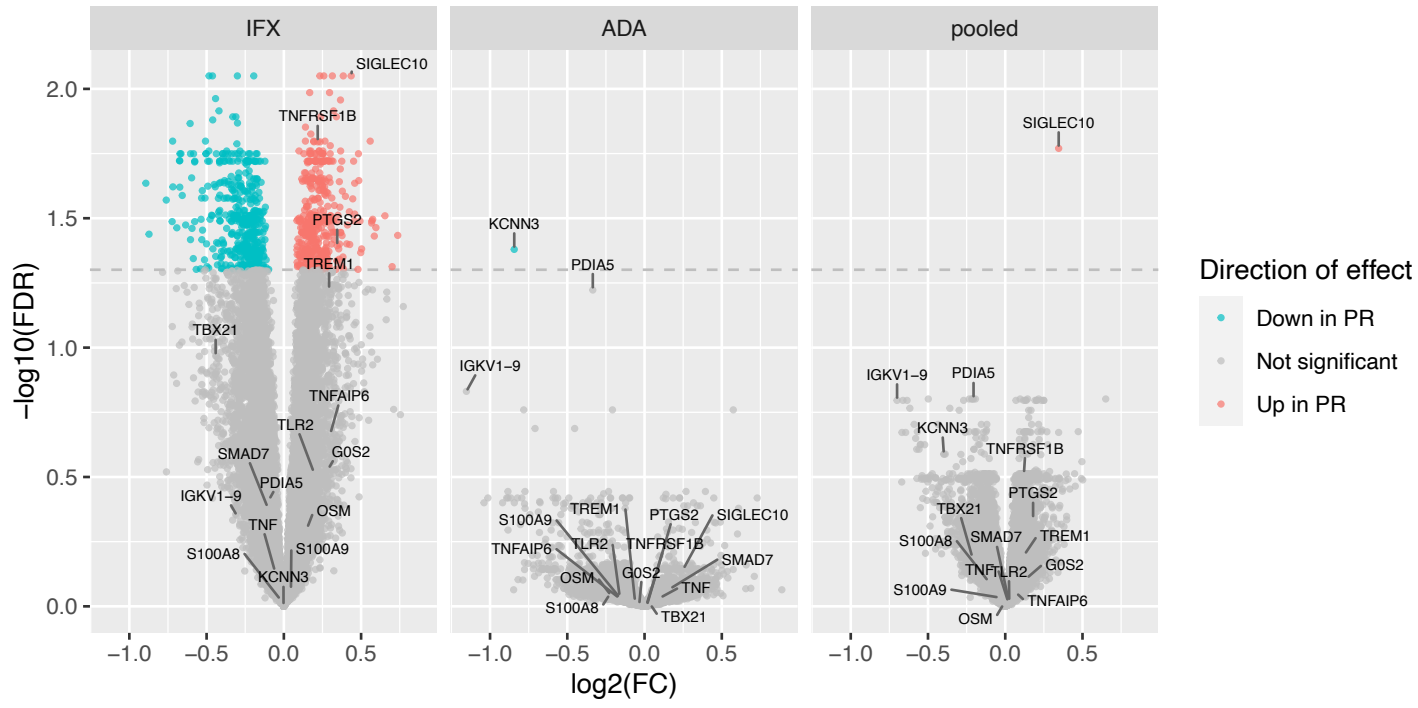

b

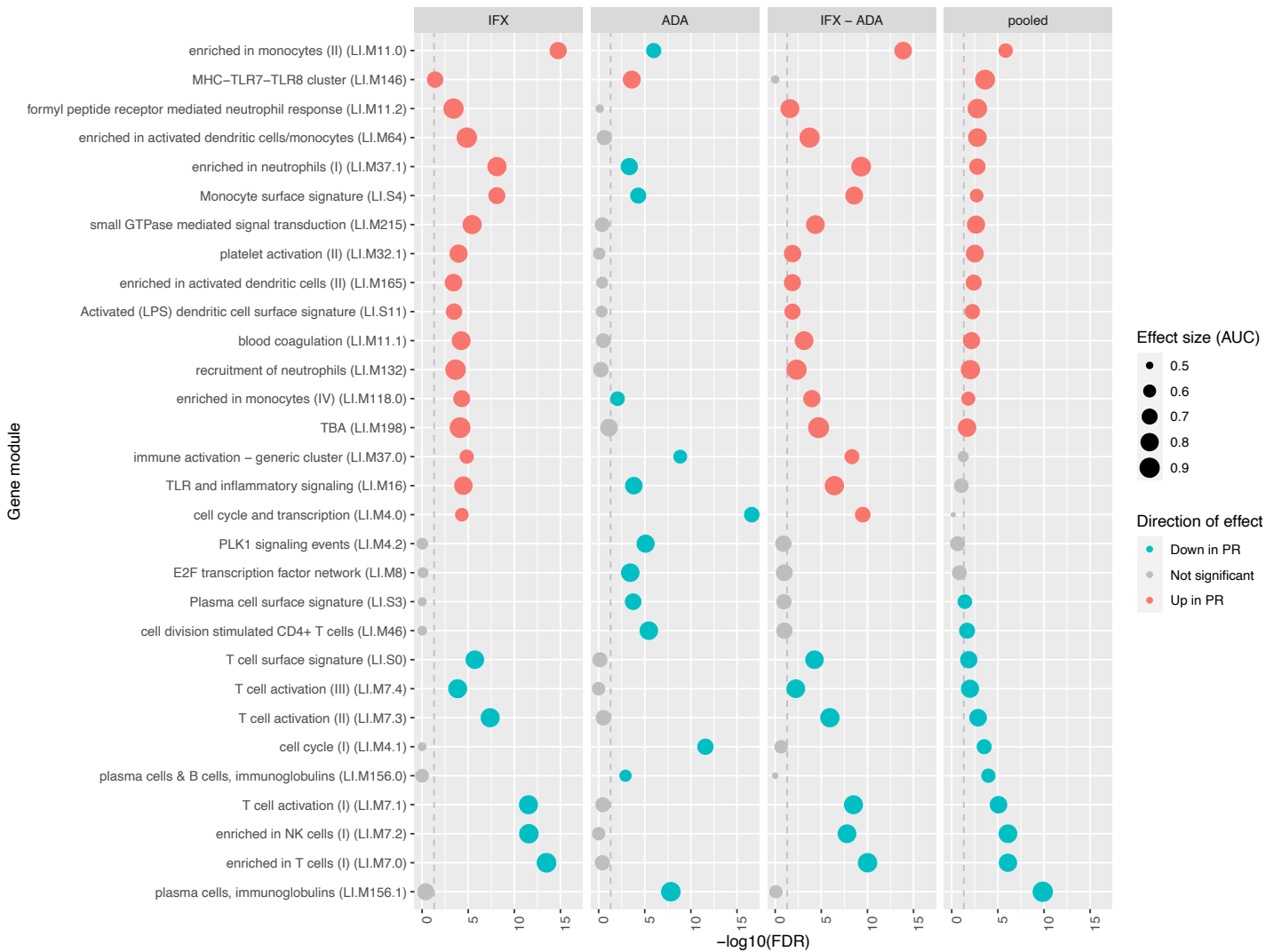

Supplement: jjad166_suppl_Supplementary_Figure_S3 [file jjad166_suppl_supplementary_figure_s3.pdf]

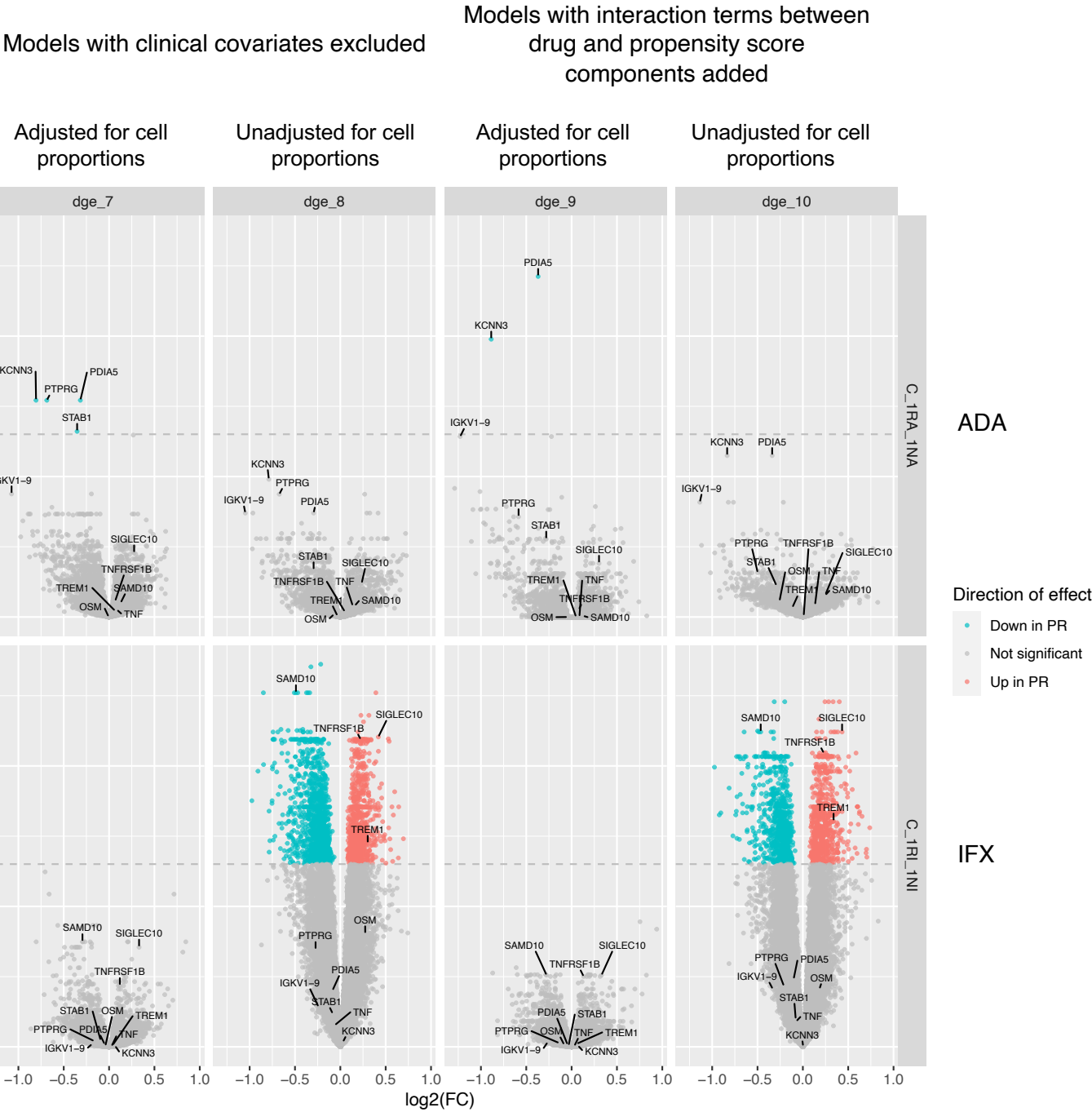

Supplement: jjad166_suppl_Supplementary_Figure_S4 [file jjad166_suppl_supplementary_figure_s4.pdf]

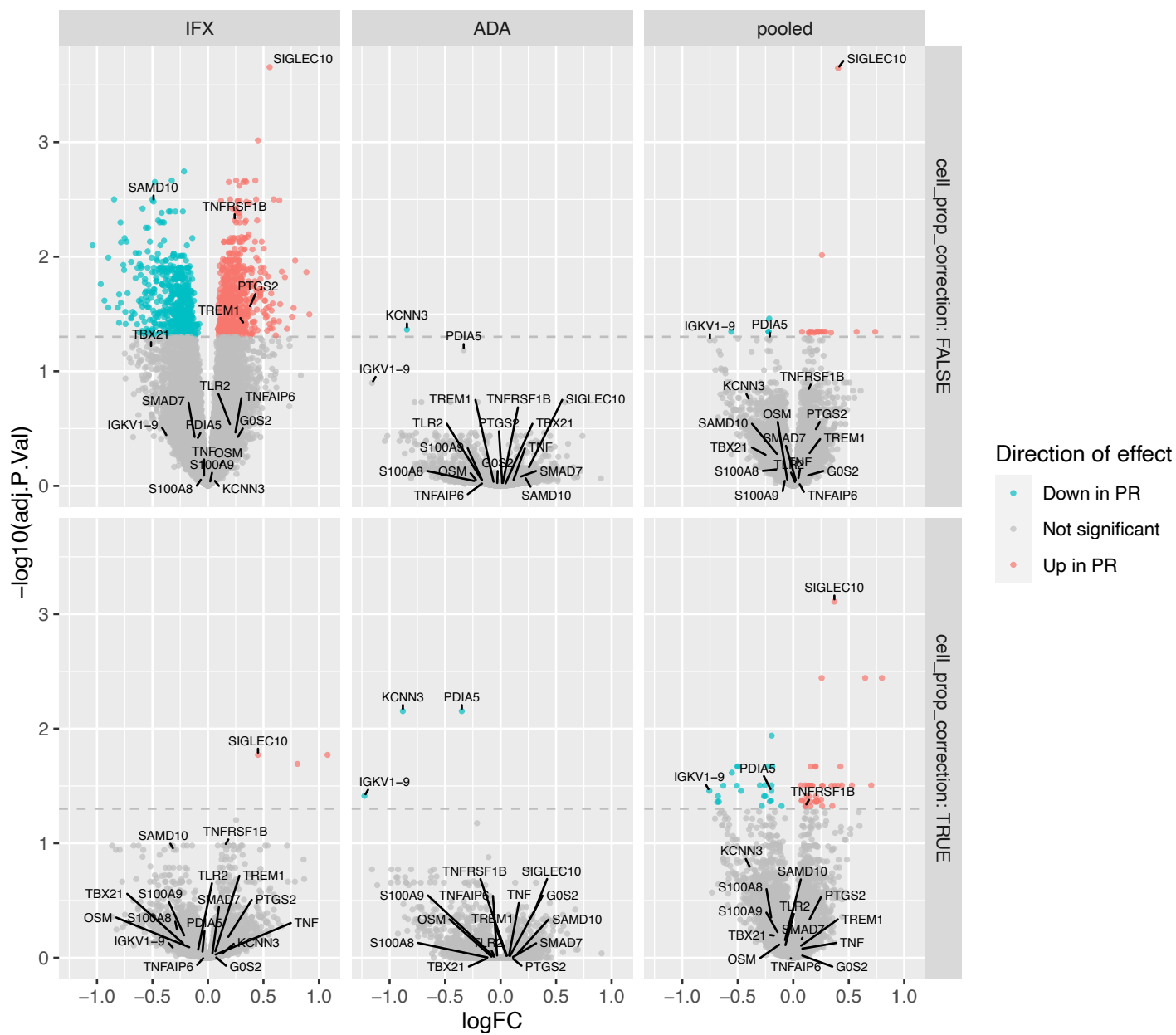

Supplement: jjad166_suppl_Supplementary_Figure_S5 [file jjad166_suppl_supplementary_figure_s5.pdf]

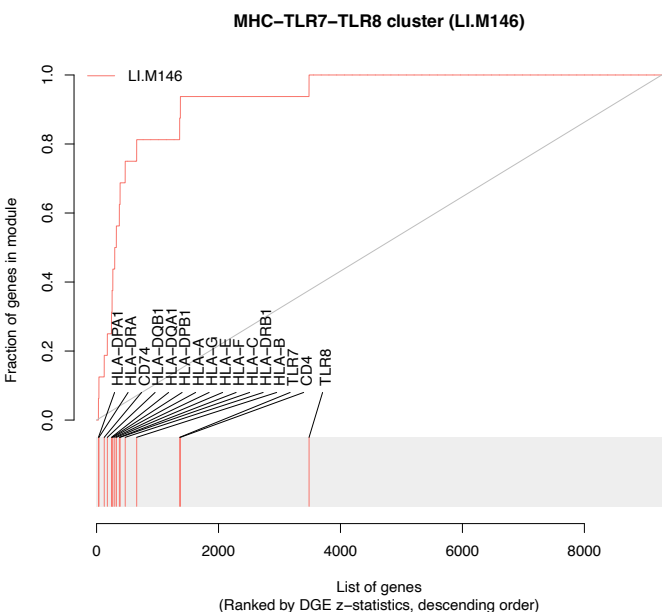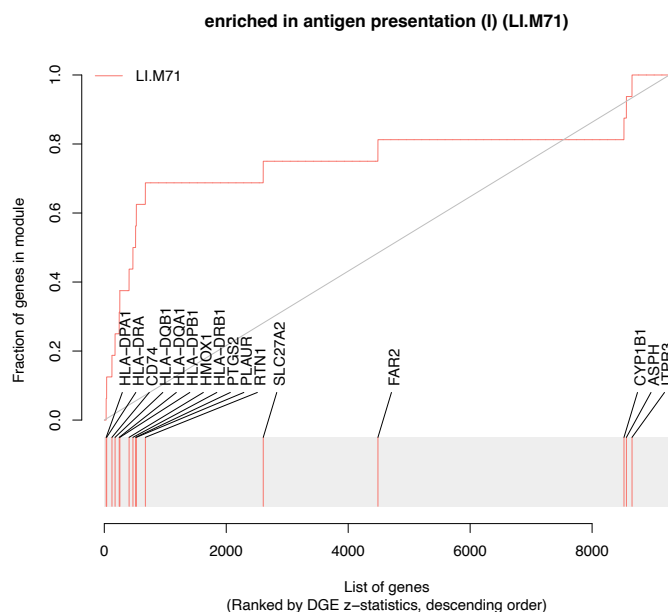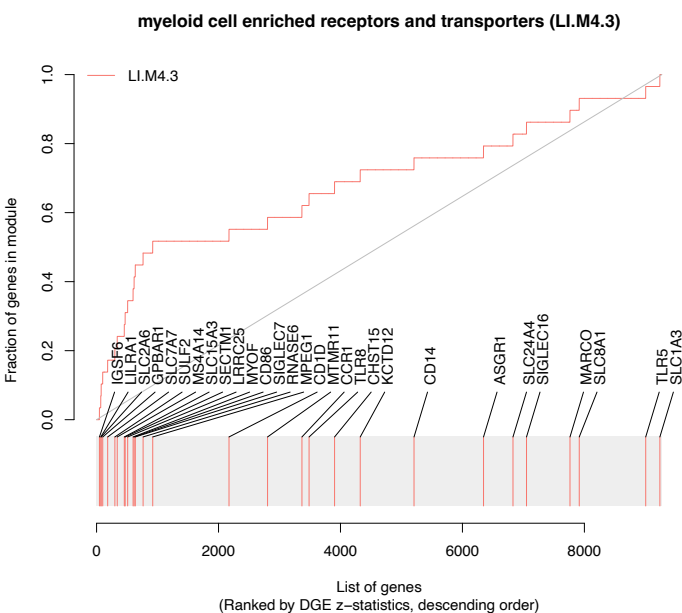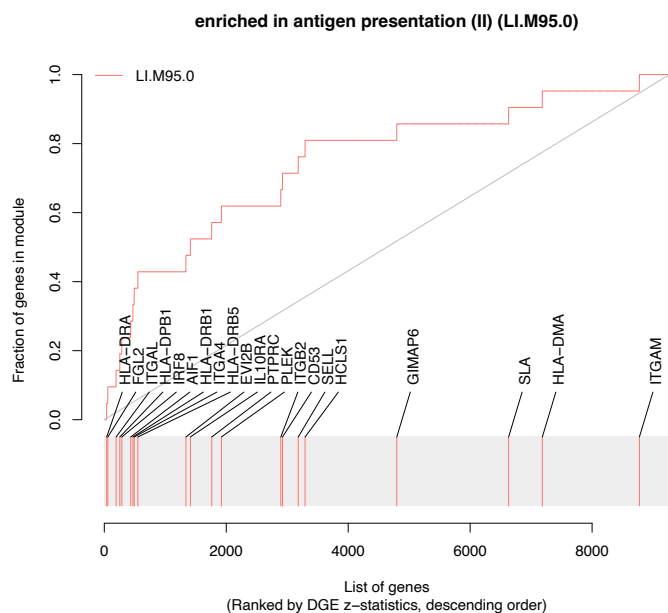

Supplement: jjad166_suppl_Supplementary_Figure_S6 [file jjad166_suppl_supplementary_figure_s6.pdf]

a

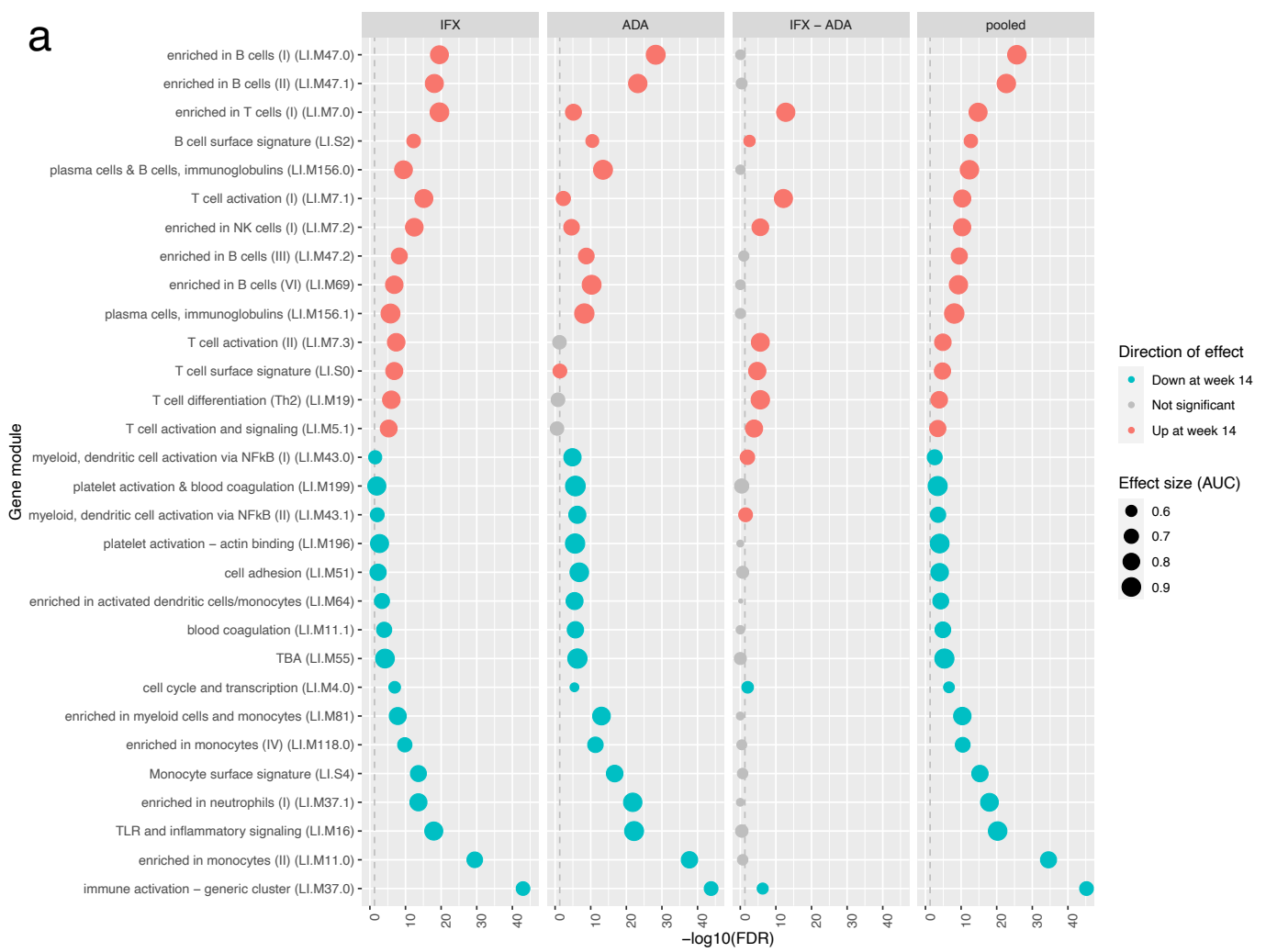

b

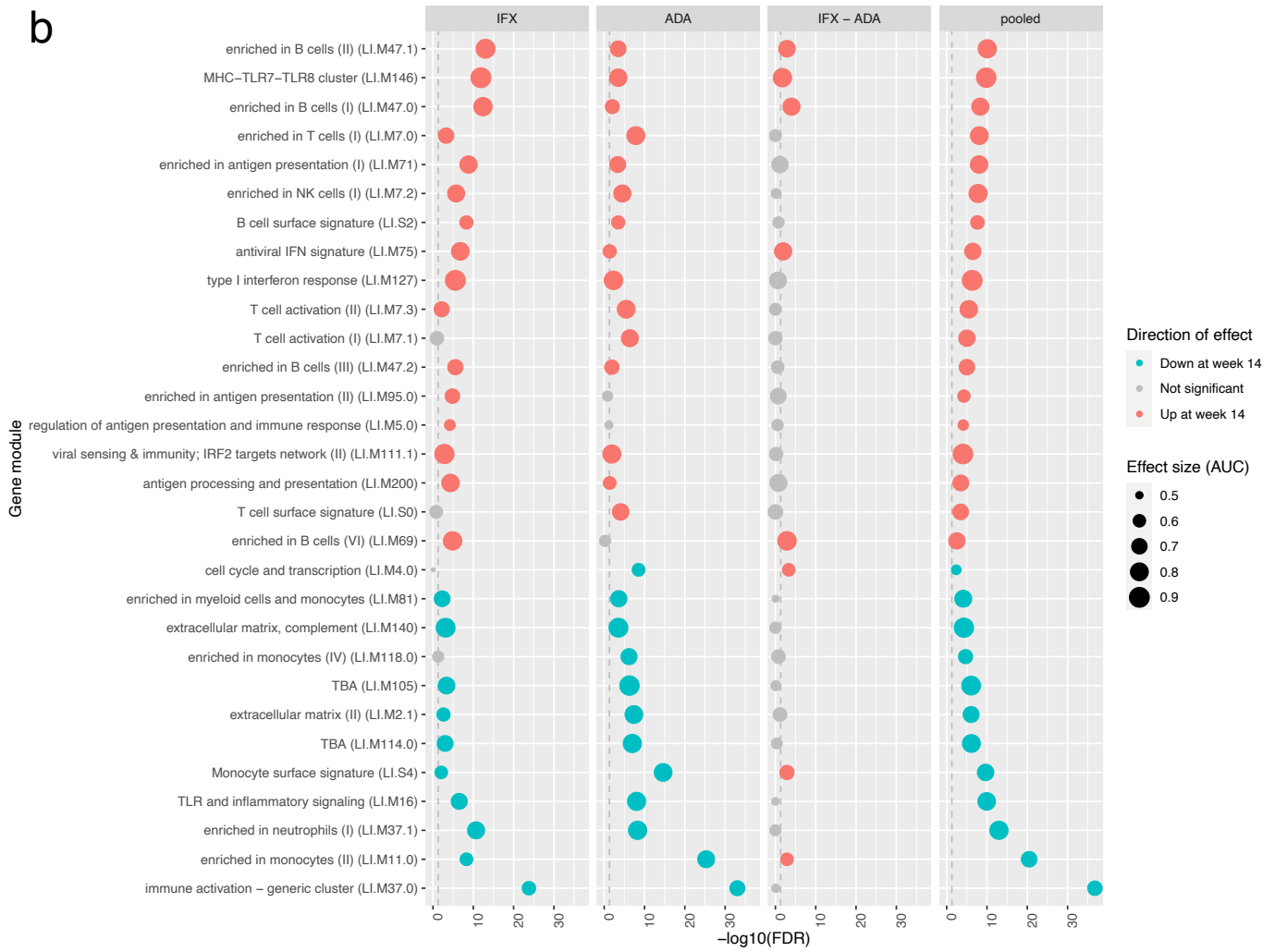

Supplement: jjad166_suppl_Supplementary_Figure_S7 [file jjad166_suppl_supplementary_figure_s7.pdf]

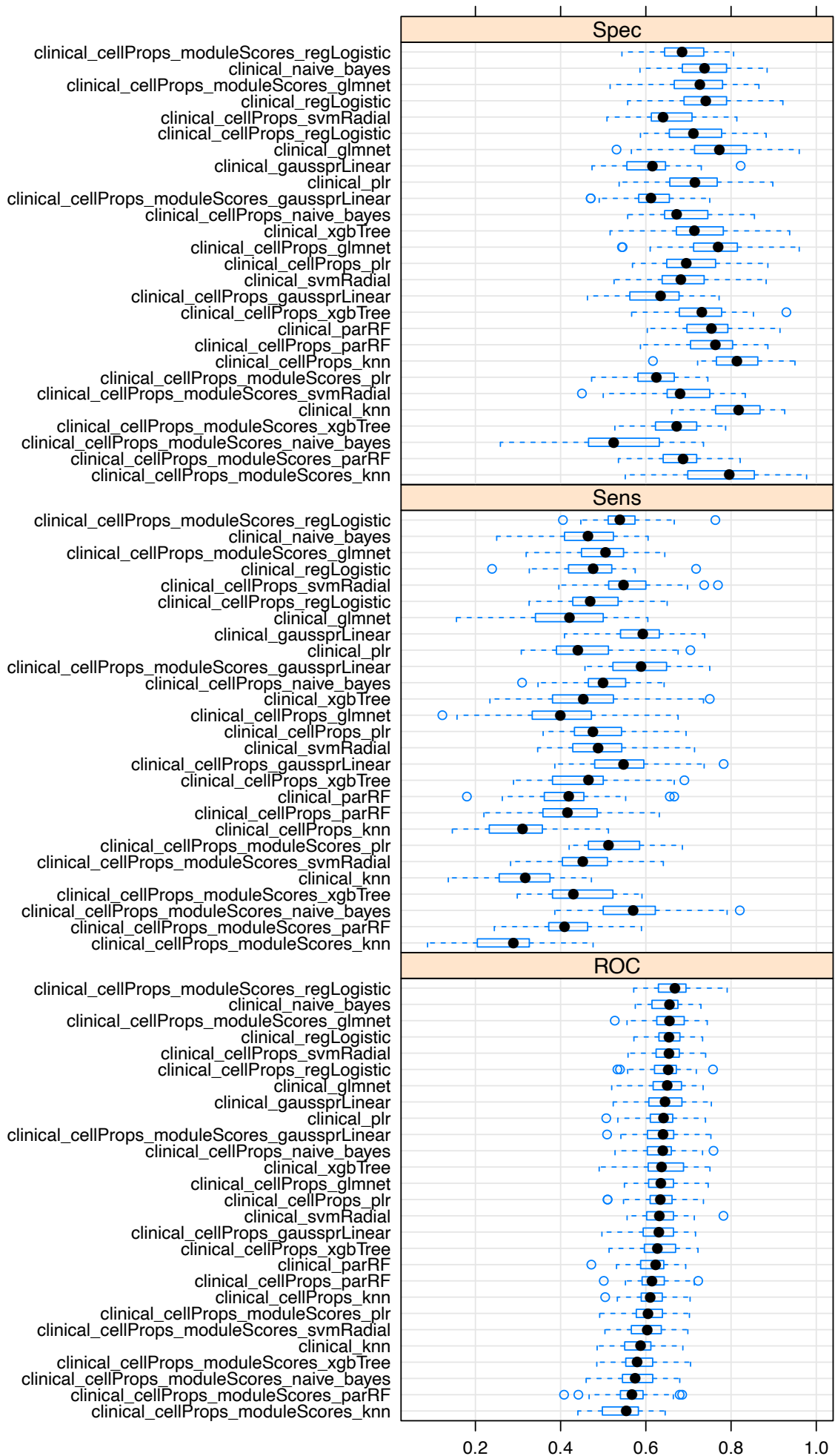

Supplement: jjad166_suppl_Supplementary_Figure_S8 [file jjad166_suppl_supplementary_figure_s8.pdf]

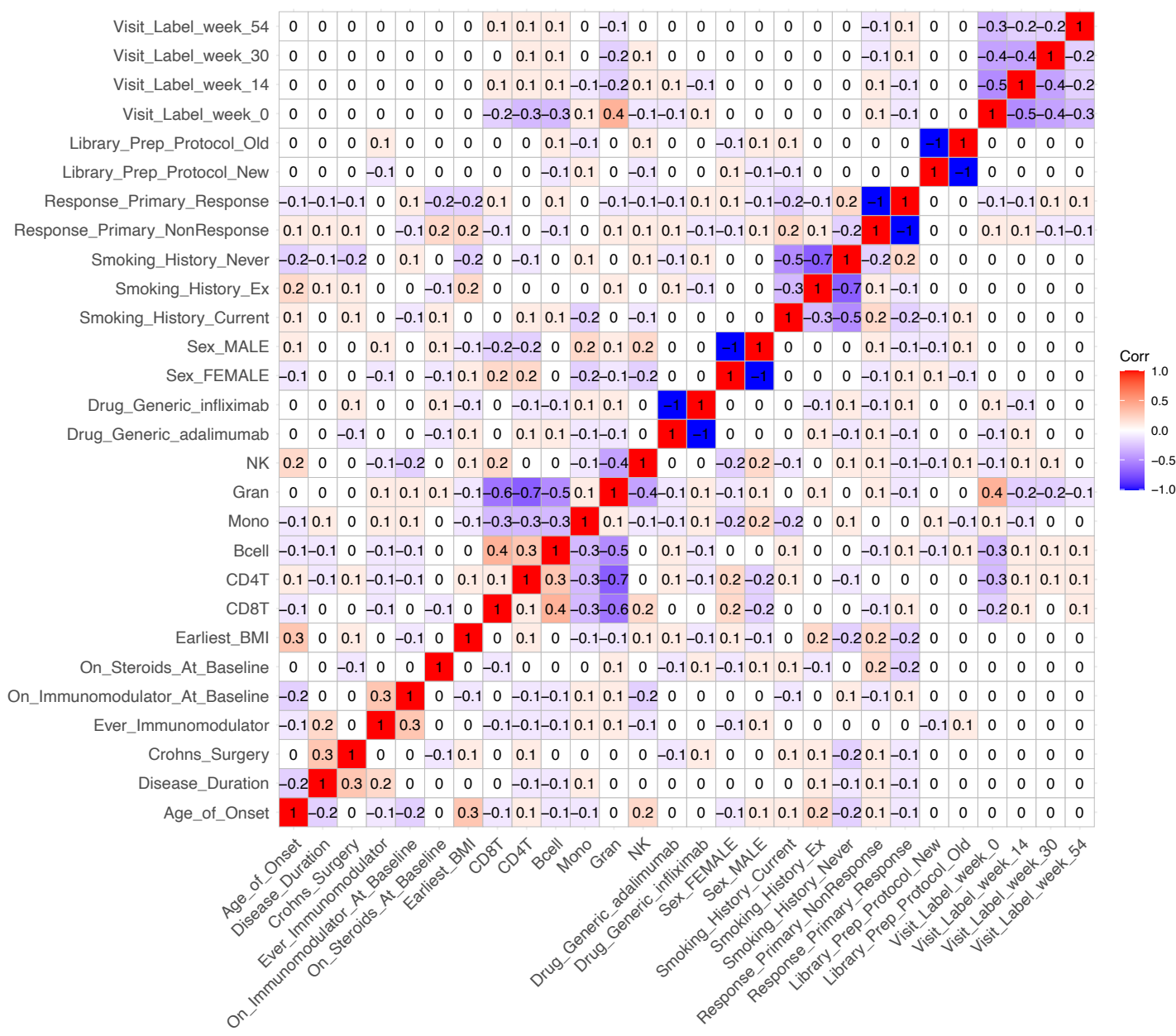

Supplement: jjad166_suppl_Supplementary_Figure_S10 [file jjad166_suppl_supplementary_figure_s10.pdf]

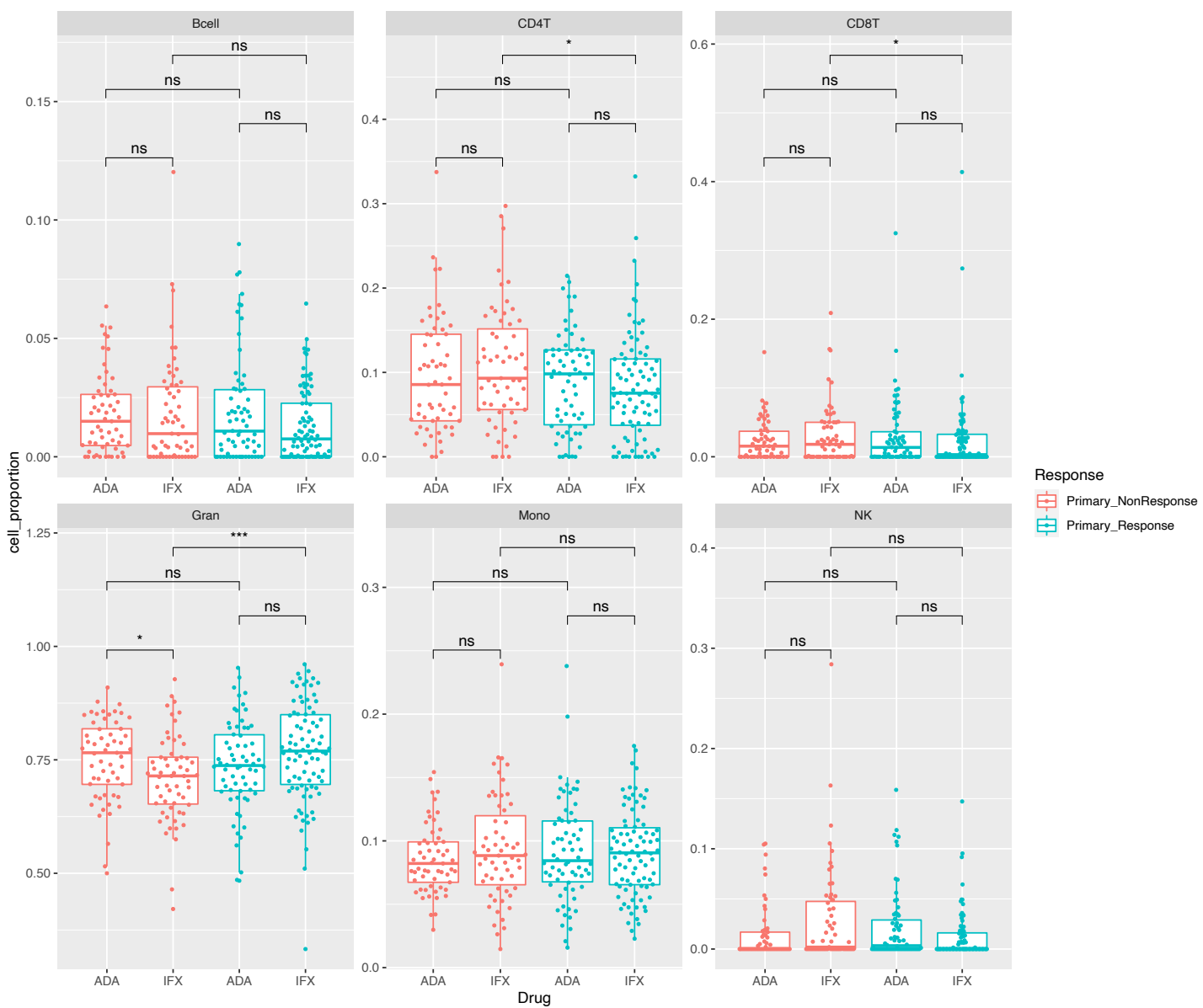

Supplement: jjad166_suppl_Supplementary_Figure_S11 [file jjad166_suppl_supplementary_figure_s11.pdf]
