# Supplementary material for: Baseline Expression of Immune Gene Modules in Blood is Associated With Primary Response to Anti-TNF Therapy in Crohn’s Disease Patients
Source: J Crohns Colitis. 2023 Sep 30;18(3):431–45. doi: 10.1093/ecco-jcc/jjad166 (PMC10906954; doi:10.1093/ecco-jcc/jjad166)
Supplement: jjad166_suppl_Supplementary_Figure_S9 [file jjad166_suppl_supplementary_figure_s9.pdf]

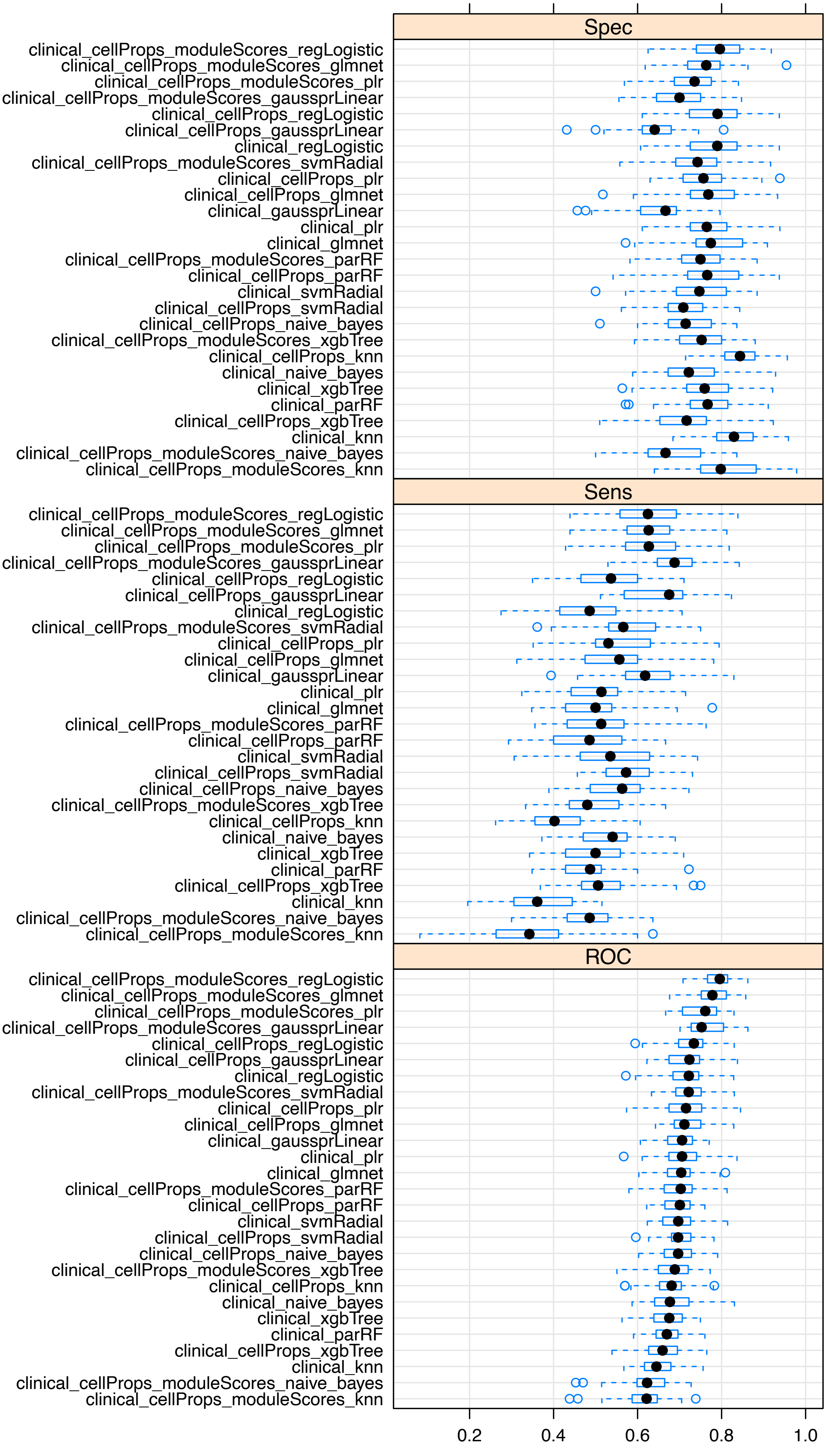
Spec
clinical_cellProps_moduleScores_regLogistic
clinical_cellProps_moduleScores_glmnet
clinical_cellProps_moduleScores_plr
clinical_cellProps_moduleScores_gaussprLinear
clinical_cellProps_regLogistic
clinical_cellProps_gaussprLinear
clinical_regLogistic
clinical_cellProps_moduleScores_svmRadial
clinical_cellProps_plr
clinical_cellProps_glmnet
clinical_gaussprLinear
clinical_plr
clinical_glmnet
clinical_cellProps_moduleScores_parRF
clinical_cellProps_parRF
clinical_svmRadial
clinical_cellProps_svmRadial
clinical_cellProps_naive_bayes
clinical_cellProps_moduleScores_xgbTree
clinical_cellProps_knn
clinical_naive_bayes
clinical_xgbTree
clinical_parRF
clinical_cellProps_xgbTree
clinical_knn
clinical_cellProps_moduleScores_naive_bayes
clinical_cellProps_moduleScores_knn
Sens
clinical_cellProps_moduleScores_regLogistic
clinical_cellProps_moduleScores_glmnet
clinical_cellProps_moduleScores_plr
clinical_cellProps_moduleScores_gaussprLinear
clinical_cellProps_regLogistic
clinical_cellProps_gaussprLinear
clinical_regLogistic
clinical_cellProps_moduleScores_svmRadial
clinical_cellProps_plr
clinical_cellProps_glmnet
clinical_gaussprLinear
clinical_plr
clinical_glmnet
clinical_cellProps_moduleScores_parRF
clinical_cellProps_parRF
clinical_svmRadial
clinical_cellProps_svmRadial
clinical_cellProps_naive_bayes
clinical_cellProps_moduleScores_xgbTree
clinical_cellProps_knn
clinical_naive_bayes
clinical_xgbTree
clinical_parRF
clinical_cellProps_xgbTree
clinical_knn
clinical_cellProps_moduleScores_naive_bayes
clinical_cellProps_moduleScores_knn
ROC
clinical_cellProps_moduleScores_regLogistic
clinical_cellProps_moduleScores_glmnet
clinical_cellProps_moduleScores_plr
clinical_cellProps_moduleScores_gaussprLinear
clinical_cellProps_regLogistic
clinical_cellProps_gaussprLinear
clinical_regLogistic
clinical_cellProps_moduleScores_svmRadial
clinical_cellProps_plr
clinical_cellProps_glmnet
clinical_gaussprLinear
clinical_plr
clinical_glmnet
clinical_cellProps_moduleScores_parRF
clinical_cellProps_parRF
clinical_svmRadial
clinical_cellProps_svmRadial
clinical_cellProps_naive_bayes
clinical_cellProps_moduleScores_xgbTree
clinical_cellProps_knn
clinical_naive_bayes
clinical_xgbTree
clinical_parRF
clinical_cellProps_xgbTree
clinical_knn
clinical_cellProps_moduleScores_naive_bayes
clinical_cellProps_moduleScores_knn
0.2
0.4
0.6
0.8
1.0
